# Model-implied simulation-based power estimation for correctly specified and distributionally misspecified models: Applications to nonlinear and linear structural equation models

**DOI:** 10.3758/s13428-024-02507-z

**Published:** 2024-10-01

**Authors:** Julien P. Irmer, Andreas G. Klein, Karin Schermelleh-Engel

**Affiliations:** Institute of Psychology, Department of Research Methods and Evaluation, Goethe University Frankfurt, Theodor-W.-Adorno-Platz 6, 60629 Frankfurt am Main, Germany

**Keywords:** Simulation, Power, Normality of parameter estimates, Model misspecification, Distributional misspecification, SEM, Moderation

## Abstract

Closed-form (asymptotic) analytical power estimation is only available for limited classes of models, requiring correct model specification for most applications. Simulation-based power estimation can be applied in almost all scenarios where data following the model can be estimated. However, a general framework for calculating the required sample sizes for given power rates is still lacking. We propose a new model-implied simulation-based power estimation (MSPE) method for the *z*-test that makes use of the asymptotic normality property of estimates of a wide class of estimators, the ***M***-estimators, and give theoretical justification for the approach. ***M***-estimators include maximum-likelihood, least squares estimates and limited information estimators, but also estimators used for misspecified models, hence, the new simulation-based power modeling method is widely applicable. The MSPE employs a parametric model to describe the relationship between power and sample size, which can then be used to determine the required sample size for a specified power rate. We highlight its performance in linear and nonlinear structural equation models (SEM) for correctly specified models and models under distributional misspecification. Simulation results suggest that the new power modeling method is unbiased and shows good performance with regard to root mean squared error and type I error rates for the predicted required sample sizes and predicted power rates, outperforming alternative approaches, such as the naïve approach of selecting a discrete selection of sample sizes with linear interpolation of power or simple logistic regression approaches. The MSPE appears to be a valuable tool to estimate power for models without an (asymptotic) analytical power estimation.

The importance of the analysis of statistical power in study planning has increased significantly in quantitative research in recent years. Assessing whether a study has sufficient power to detect a specific effect size is essential for study planning (e.g., see Cohen, 1988, 1992). In statistical testing, for a given significance level $\alpha$, statistical power is defined as the probability of correctly rejecting a null hypothesis ($H_0$) if the alternative hypothesis ($H_1$) is true for the population.

Power may be estimated globally or locally, depending on the specific hypotheses being tested. Assessing the power of overall model characteristics, such as model fit, is summarized under the term *global* power analysis (see, e.g., Jobst, Bader, & Moshagen, 2023). Of interest within *local* power analysis is the significance of individual parameters within the model. In this article, we focus on local power estimation for the expected effect size of individual parameters.

Two main classes of power analysis procedures are commonly distinguished: purely analytical, but often asymptotic, power procedures and Monte Carlo simulation-based power estimation techniques (Feng & Hancock, 2023). Analytical power estimation uses mathematical theorems and asymptotics to derive power. This enables solving for the required sample size *n*. Simulation-based power estimation generally involves generating data for a chosen model *R* times across preselected sample sizes. For each preselected sample size, power is assessed by calculating the relative frequency of a significant test result (e.g., Muthén & Muthén 2002).

Both approaches find wide application in research, with implementations available in various software packages. One of the most widely used analytical power estimation software packages is G*Power (Faul, Erdfelder, Lang, & Buchner, 2007, 2009). The pwr package (Champely, 2020) and WebPower (Zhang & Yuan, 2018) offer implementations for power analysis of many basic statistical tests in R (R Core Team, 2023), with extensions also available for linear structural equation models (SEM, see also Moshagen & Bader, 2024, for an implementation). (Asymptotic) analytical power estimation procedures offer the advantage of being free from sampling error and, in most cases, having a low computational burden, making them particularly useful when available.

Simulation-based power estimation methods have gained popularity in applied research, particularly for methods lacking analytical power solutions. Simulation-based power estimation is applicable in most scenarios where data can be simulated according to a predefined model. The ability to incorporate specific population characteristics into the simulation, such as distributional misspecifications, and the ease with which these simulations can often be implemented, make this approach particularly appealing. In the social sciences, several developments have occurred in R, including hierarchical regression models (Kumle, Võ, & Draschkow, 2021), linear SEM (Wang & Rhemtulla, 2021), and special classes of linear SEM, such as random-intercept cross-lagged panel designs (powriclpm package, Mulder, 2023), and moderated SEM with categorical moderators (simsem package, Pornprasertmanit, Miller, Schoemann, & Jorgensen, 2021).

For these simulation-based power estimation procedures, the relationships between power estimates and sample size are often linearly interpolated (connected by straight lines) to graph the overall dependency between statistical power and sample size *n* (see, e.g., Mulder, 2023; Wang & Rhemtulla, 2021, for examples of this procedure). The sample sizes used for power estimation are often selected in a discrete and ad hoc manner. Similar limitations hold for the selection of the number of replications. The estimated power for a given *n* has high precision only for the selected sample sizes but lacks precision when linear interpolation is used for samples in between. Thus, linear interpolation can introduce bias when estimating power between selected sample sizes and does not allow for extrapolation of power rates. To overcome this limitation, Schoemann, Miller, Pornprasertmanit, and Wu (2014) used a logistic regression model to predict power within linear SEM as a continuous function.

In this paper, to address the limitations associated with the ad-hoc selection of sample size and the linear interpolation of the power curve between selected sample sizes, we propose a novel model-implied simulation-based technique to estimate power for the (single-parameter) *z*-test. It makes particular use of the distributional characteristics of the parameter estimates. Our approach aims to better approximate the power curve of the *z*-test by using a probit regression model. The approach goes beyond the work of Schoemann et al. (2014), who utilized a logit link to estimate power in simulation procedures for linear SEM. We expand upon their idea in three ways: First, we make use of the distribution characteristics of the parameter estimates and apply it to provide a justification for the choice of a link function. Second, going beyond the work of Schoemann et al. (2014), we invert the link function and derive the required sample size given a certain effect size. In simulations, also the inverted power curve underlies a sampling variation. Therefore, third, we include this aspect in our procedure for an optimized calculation of required sample size. These enhancements improve the accuracy (unbiased estimates for power and, therefore, for required samples sizes) and efficiency (smaller confidence intervals for power and, therefore, smaller variance in required sample sizes) of power estimation in our proposed method, which is especially important for time-consuming estimation methods.

Altogether, the aim of this paper is to develop a simulation-based power estimation method that continuously describes the relationship between power and sample size for the *z*-test using a parametric model. This approach efficiently utilizes the statistical information from all simulated data and can be applied to determine the required sample size. Hence, the aims of this article are threefold. First, we propose the new model-implied simulation-based power estimation (MSPE) method and provide its necessary theoretical justifications, second, we illustrate its application for two different model types: linear SEM and quadratic and interaction SEM (QISEM), for correctly specified models and, third, we demonstrate the usefulness of our proposed method under distributional misspecification. Finally, advantages and limitations of MSPE are discussed.

## Power estimation utilizing the distributional information of the parameter estimates

In this section, we propose a model-implied simulation-based power estimation (MSPE) method for the *z*-test that leverages the asymptotic normality of a wide class of estimators.

In simulation-based power estimation, datasets are simulated *R* times from a target model for various sample sizes. Subsequently, the effects of interest are evaluated for significance using the *z*-test, and the relative frequency of significant results is taken as an estimate for statistical power. However, it is important to note that the estimated power is only accurate for the selected sample sizes.

The precision of the simulated power curve of the *z*-test depends on the number of replications selected. The choice of sample sizes becomes especially crucial if the method is time-consuming, as may be the case for complex models like nonlinear SEM. In our method, we utilize correlational information between power rate and sample size by fitting a parametric model for the power curve of the *z*-test. This approach, unlike point-wise power analysis for selected sample sizes, results in substantially narrower confidence intervals around the power curve.

### Ad hoc power estimation of significance decisions

Instead of selecting sample sizes and using linear interpolation among them, Schoemann et al. (2014) fitted a logit-regression model to describe the continuous relation between power rates of the *z*-test and *n*. However, Schoemann et al. (2014) provided no particular justification for the use of *n* as a predictor in a logistic model for the power curve. Nor did they make any use of the distribution characteristics of the parameter estimation within the logistic regression to improve the procedure. In this paper, we go beyond their work by considering the asymptotic normality of a wide class of estimators, refine the relationship between sample size and power, and further invert the implied power to solve for sample size and describe our idea and the consequences for modeling power.

### Distribution-based analysis of statistical power

Let $\vartheta$ be the parameter vector of the model to be estimated. Consider estimating the population parameter vector $\vartheta _0$ of the model by $\hat{\vartheta }_n$ for a sample of size *n*. For a very general class of estimators, the *M*-estimators (Wooldrige, 2010, Chapter 12), the estimates $\hat{\vartheta }_n$ are asymptotically normal under certain regularity conditions, which are generally met in applied research, when all parameters of the model are identified. Formally, this is denoted by

1 $$\begin{aligned} \sqrt{n} (\hat{\vartheta }_n - \vartheta _0) \overset{d}{\underset{n\rightarrow \infty }{\longrightarrow }}\mathcal {N}\left( 0, \mathcal {H}^{-1}\mathcal {I} \mathcal {H}^{-1}\right) , \end{aligned}$$

where $\overset{d}{\underset{n\rightarrow \infty }{\longrightarrow }}$ denotes convergence in distribution and $\mathcal {N}\left( 0, \mathcal {H}^{-1}\mathcal {I} \mathcal {H}^{-1}\right)$ is the multivariate normal distribution with zero mean and covariance matrix $\mathcal {H}^{-1}\mathcal {I} \mathcal {H}^{-1}$. The Fisher-Information matrix, also called the variance of the score function, is denoted by $\mathcal {I}$ and the Hessian of the objective function (e.g., of the log-likelihood) is denoted by $\mathcal {H}$, evaluated at the true parameter vector. From $\mathcal {I}$ and $\mathcal {H}$, the asymptotic covariance matrix of the estimates is derived by

$$AVAR(\hat{\vartheta }_n) = \frac{1}{n}\mathcal {H}^{-1}\mathcal {I} \mathcal {H}^{-1}, $$

where the square root of the diagonal elements are the standard errors (see, e.g., Huber, 1967; White, 1980, 1982; Wooldrige, 2010, Chapter 12). Special cases of *M*-estimators are ML, quasi-ML (e.g., White, 1982), ordinary and weighted least squares (OLS, WLS), nonlinear least squares (NLS, e.g., Wooldrige, 2010, Chapter 12) and (generalized) method of moments (MM, GMM, e.g., Hansen, 1982) estimators (Wooldrige, 2010, Chapter 12). Since *M*-estimators are not restricted to ML-estimators, which require the correctly specified distribution of the data, models including distributional or model misspecification are also included (e.g., White, 1980, 1982). Consequently, many estimators used in applied research fall in this class. For finite samples $\hat{\vartheta }_n$ is approximately normally distributed with expectancy $\mathbb {E}\left[ \hat{\vartheta }_n\right] =\vartheta _0$ for large *n*. Let $\hat{\vartheta }_{n,j}$ be the *j*-th entry of $\hat{\vartheta }_n$. The standard error of $\hat{\vartheta }_{n,j}$ is denoted as $SE\left( \hat{\vartheta }_{n,j}\right)$. Hence, under the null hypothesis $H_0: \vartheta _{0,j}=0$, the *z*-value $\mathcal {Z}^{H_0}_n$ of the estimate typically returned by statistical software is given by

2 $$\begin{aligned}&\mathcal {Z}^{H_0}_n = \frac{\hat{\vartheta }_{n,j}}{SE(\hat{\vartheta }_{n,j})}, \quad \mathcal {Z}^{H_0}_n \overset{a}{\sim }\mathcal {N}(0,1), \nonumber \\&\quad SE\left( \hat{\vartheta }_{n,j}\right) =\sqrt{\frac{1}{n}\left( \mathcal {H}^{-1}\mathcal {I}\mathcal {H}^{-1}\right) _{j,j}}, \end{aligned}$$

where $\overset{a}{\sim }$ denotes approximate distribution meaning that for large sample sizes (*n*) the *z*-value $\mathcal {Z}^{H_0}_n$ should be close to a standard normal variable in terms of the distribution. Consequently, for a predefined type I error rate $\alpha$, asymptotically, the probability of a significant result denoted by the event $\{\mathcal {Z}^{H_0}_n>q_\text (1-\alpha )\}$ is $\alpha$, where $q_\text (1-\alpha )$ is the $1-\alpha$ quantile of the standard normal distribution.

Under the alternative hypothesis $H_1$ (i.e., $\vartheta _{0,j}\ne 0$), the *z*-value no longer has zero mean. Instead, the mean of the *z*-value under $H_1$ is proportional to the true effect size $\vartheta _{0,j}$ divided by the standard error $SE\left( \hat{\vartheta }_{n,j}\right)$

3 $$\begin{aligned} \mathcal {Z}^{H_1}_n=\frac{\hat{\vartheta }_{n,j}}{SE(\hat{\vartheta }_{n,j})}, \quad \mathcal {Z}^{H_1}_n \overset{a}{\sim }\mathcal {N}\left( \frac{\vartheta _{0,j}}{SE(\hat{\vartheta }_{n,j})},1\right) . \end{aligned}$$

Hence, the mean of the *z*-value under $H_1$ increases indefinitely for increasing *n*

4 $$\begin{aligned} \mathbb {E}\left[ \mathcal {Z}^{H_1}_n \right] = \frac{\vartheta _{0,j}}{SE(\hat{\vartheta }_{n,j})} = \sqrt{n}\cdot \frac{\vartheta _{0,j}}{\sqrt{\left( \mathcal {H}^{-1}\mathcal {I}\mathcal {H}^{-1}\right) _{j,j}}} \underset{n\rightarrow \infty }{\longrightarrow }\ \pm \infty , \end{aligned}$$

since $\frac{\vartheta _{0,j}}{\sqrt{\left( \mathcal {H}^{-1}\mathcal {I}\mathcal {H}^{-1}\right) _{j,j}}}$ is constant. Consequently, the probability of a significant parameter converges to 1 for increasing sample size (see Appendix A for more details).

### Proposition of the new model-implied simulation-based power estimation method

Now we use the described properties of the parameter estimates typically used in applied research to propose a model for power estimation for the *z*-test using the asymptotic normality properties of the parameter estimates. Here, we analyze a one-sided test, where we assume that the hypothesis for direction is correct. However, the results also hold for a two-sided test (see Appendix B). For a positive population effect, a significant result is observed if the *z*-value exceeds the threshold $q_\text (1-\alpha )$, while for a negative population effect, a significant result is observed if the *z*-value has a largely negative value and falls below the lower bound $q_\text (\alpha )$. Formally, a significant event for $\vartheta _{0,j}>0$ is given by $\left\{ \mathcal {Z}^{H_1}_n>q_\text (1-\alpha )\right\}$ and for $\vartheta _{0,j}<0$ a significant event is given by $\left\{ \mathcal {Z}^{H_1}_n<q_\text (\alpha )\right\}$ $= \left\{ -\mathcal {Z}^{H_1}_n>q_\text (1-\alpha )\right\}$. Hence, we can state a general significant event by simply multiplying the *z*-value with the sign ($sign(\vartheta _{0,j})=\pm 1$) of the population effect

$$ \left\{ (sign(\vartheta _{0,j})\mathcal {Z}^{H_1}_n>q_\text (1-\alpha )\right\} . $$

For further justifications and derivations see Appendix A.

As the asymptotic distribution and its mean of the *z*-value is known under the alternative hypothesis $H_1$, we make use of Eqs. 3 and 4 in order to describe the probability of the event $\left\{ sign(\vartheta _{0,j})\mathcal {Z}^{H_1}_n>q_\text (1-\alpha )\right\}$ by approximating it with the value of the distribution function $\Phi$ of the standard normal distribution

5 $$\begin{aligned}&\mathbb {P}\left( sign(\vartheta _{0,j})\mathcal {Z}^{H_1}_n>q_\text (1-\alpha )\right) \nonumber \\&\quad \approx \Phi \left( -q_\text (1-\alpha )+\frac{\left| \vartheta _{0,j} \right| }{\sqrt{\left( \mathcal {H}^{-1}\mathcal {I}\mathcal {H}^{-1}\right) _{j,j}}}\sqrt{n}\right) . \end{aligned}$$

Let $\mathcal {S}_i$ be the indicator which codes the significance decision for $i=1,\dots ,R$ simulations, i.e., $\mathcal {S}_i=1$ if the *i*-th replication resulted in a significant result and $\mathcal {S}_i=0$ else. Then the power curve is fitted by a probit regression model

6 $$\begin{aligned}&\mathbb {P}\left( \mathcal {S}_i = 1 | n_i, \beta _0, \beta _1 \right) = \Phi (\beta _0 + \beta _1 \sqrt{n_i}), \nonumber \\&\quad \beta _\text {probit}=(\beta _0,\beta _1)'=\left( -q_\text (1-\alpha ),\frac{\left| \vartheta _{0,j}\right| }{\sqrt{\left( \mathcal {H}^{-1}\mathcal {I}\mathcal {H}^{-1}\right) _{j,j}}}\right) ', \end{aligned}$$

for $i=1,\dots ,R$. Hence, the significance decision follows a probit model with the square root of the sample size ($\sqrt{n}$) as a predictor, where the asymptotic parameters (large *n* and $R\rightarrow \infty$) of the probit regression are denoted by $\beta _\text {probit}$. These probit regression parameters are used to calculate the required sample size $N_\alpha$ for a predefined power rate $\rho$ by inverting the link function in Eq. 5 and solving for sample size.

7 $$\begin{aligned} N_\alpha&\approx \! \left\lceil \left( \frac{\Phi ^{-1}(\rho ) -\beta _0}{\beta _1}\right) ^2\right\rceil \nonumber \\&= \! \left\lceil \Bigg (\Phi ^{-1}(\rho ) + q_\text (1-\alpha ) \Bigg )^2 \cdot \left( \frac{|\vartheta _{0,j}|}{\sqrt{\left( \mathcal {H}^{-1}\mathcal {I}\mathcal {H}^{-1}\right) _{j,j}}}\right) ^{-2}\right\rceil , \end{aligned}$$

where $\lceil \cdot \rceil$ denotes rounding up to the next integer. For more details and derivations see Appendix B.

Schoemann et al. (2014) used a logit regression model instead of a probit regression with *n* as a predictor. However, probit and logit regression models are close and only differ to a large amount at the edges of the support (Amemiya, 1981), which is probably why it served as a crude approximation of power (Schoemann et al., 2014). We now extend the work of Schoemann et al. (2014) by using the standard errors for the implied power by the probit (or logit) regression model when optimizing for the smallest *n* that ensures a certain power rate.

Simulation-based procedures are subject to sample variability that decreases with increasing *R*. In order to model the uncertainty in the parameter estimation in the probit regression, we make use of the asymptotic normality of the coefficients’ estimates within the probit regression following form ML theory to compute $1-\alpha _\rho$ confidence bands for the predicted power values. We then compute the required sample size that ensures a type I error rate to be smaller than the population sample size $N_\alpha$ of $\alpha _\rho /2$:

8 $$\begin{aligned} N_{\alpha ,\text {lb}}&\approx \left\lceil \left( \frac{\Phi ^{-1}\left( \hat{\rho }_{\hat{\rho }_\text {lb} = \rho }\right) -\beta _0}{\beta _1}\right) ^2\right\rceil , \end{aligned}$$

where $\hat{\rho }_{\hat{\rho }_\text {lb}=\rho }$ is the power rate with a corresponding lower bound of the $1-\alpha _\rho$ confidence interval to equal the selected power rate $\rho$. Then, the probability of $N_{\alpha ,\text {lb}}$ being larger or equal to $N_\alpha$ is $\approx 1-\alpha _\rho /2$.

If the expected Hessian $\mathcal {H}$ and the variance of the scores $\mathcal {I}$ are known analytically, then inputting these directly in Eq. 7 gives a close approximation for $N_\alpha$. Demidenko (2007) derived power for correctly specified ML-estimation in logistic regression with a single linear predictor. The procedure requires complex integration which can be tedious for models with many observed variables. If $\mathcal {H}$ and $\mathcal {I}$ are unknown, the estimated probit regression coefficients from *R* replications in a simulation study can be used to approximate $N_\alpha$ and $N_{\alpha ,\text {lb}}$ by replacing the coefficients $\beta _0,\beta _1$ by their estimates $\hat{\beta }_0,\hat{\beta }_1$ in Eqs. 7 and 8.

Demidenko (2007) noted, among others, that when conducting power analysis, the test for which the power is available should be equivalent to the test used in applied research. For example, although the Wald test and the likelihood ratio test are asymptotically equivalent, they differ in finite samples (Buse, 1982; Engle, 1984). The two-sided *z*-test is equivalent to the one-parameter Wald test. Both examine the event $\big \{|\mathcal {Z}^{H_1}_n|>$ $q_\text (1-\alpha /2)\big \}$. For the two-sided test and, hence, the Wald test, a version of a probit regression can be derived (see end of Appendix B). For this variant of probit regression, the MSPE is also applicable. An example application for the MSPE in comparison to linear interpolation of power values is given in Fig. 8 in Appendix D. In this example, the difference between MSPE and linear interpolation is not very large, but it is clearly present. In addition, the confidence interval of MSPE is significantly smaller than the confidence interval of linear interpolation resulting in estimates for $N_{\text {lb},\alpha }$ closer to the true required sample size. The reason for the good performance of MSPE is provided in the next sections.

## Application of the new MSPE method

In this section, we numerically illustrate the performance of the MSPE method through two simulation studies. We examine a general nonlinear model and select sub-models commonly encountered in research to assess the performance of the MSPE. For these sub-models, we consider linear SEM and QISEM, both in scenarios where the model is correctly specified and when there is distributional misspecification. All analysis scripts and additional plots can be found at https://osf.io/6qh4u/.

**Fig. 1:**
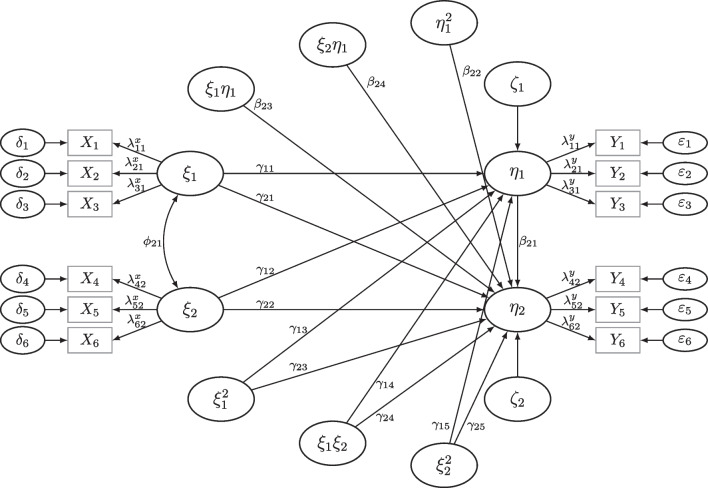
Nonlinear SEM including moderation and quadratic effects. *Note*. Mean structure and intercepts are not included in the figure. Further, model implied correlations among the terms $\xi _1\xi _2$, $\xi _1\eta _1$, and $\xi _2\eta _1$ are not displayed for clarity

The MSPE is applicable to a wide class of models. In this paper, we focus on several examples including nonlinear effects and measurement error, i.e., models typically encountered in the social sciences and related fields. For nonlinear models including measurement error, general power solutions are still missing. Nonlinear SEM (NLSEM) include such models that consist of a vector of dependent variables $\eta$ that is influenced by functions of the vector of independent variables $\xi$ given by

$$\begin{aligned} \eta = H(\xi ) + \zeta , \end{aligned}$$

for a continuous function *H*, where the residual of $\eta$ is of the same dimension and is denoted by $\zeta$ (see, e.g., Grønneberg & Irmer, 2024, where *H* is the conditional expectation $H(x) = \mathbb {E}[\eta | \xi = x]$ and $\zeta$ is the corresponding residual $\zeta = \eta - \mathbb {E}[\eta | \xi ]$). $\eta$ and $\xi$ are generally observed with measurement error via observation *Y* and *X*, respectively, where the dimensions of the measurements generally exceed the dimensions of the latent variables necessary for model identification. Although *H* can be general, large interest lies in models that include interaction effects also called moderation effects (Kenny & Judd, 1984). Such models include effects up to order 2, which is why they are called quadratic and interaction SEM (QISEM, see, e.g., Büchner & Klein, 2020). Although there are some methods that are able to estimate general nonlinear forms, such as factor score approaches, these have not been intensively studied. For instance, under some assumptions, simple factor score approaches are expected to perform well for nonlinear models as long as many measurements are used per latent variable. This can be derived from results on conditional expectations for factor scores (see, e.g., Grønneberg & Irmer, 2024) as long as the parametric form of the model is correctly specified (see, also, Wall & Amemiya, 2000, 2001). However, since QISEM have been studied extensively (e.g., Brandt, Kelava, & Klein, 2014; Brandt, Umbach, Kelava, & Bollen, 2020; Kenny & Judd, 1984; Klein & Moosbrugger, 2000; Marsh, Wen, & Hau, 2004; Ng & Chan, 2020; Wall & Amemiya, 2000, 2001) and are widely used (Aytürk, Cham, Jennings, & Brown, 2020), we focus on the estimation of power in QISEM and related models in the following. Figure 1 depicts a NLSEM with the following structural model

$$\begin{aligned} \eta _1&= \alpha _1 + \gamma _{11}\xi _1 + \gamma _{12}\xi _2 + \gamma _{13}\xi _1^2 + \gamma _{14}\xi _1\xi _2 + \gamma _{15}\xi _2^2 + \zeta _1,\\ \eta _2&= \alpha _2 + \beta _{21}\eta _1 + \beta _{22}\eta _1^2 + \beta _{23}\eta _1\xi _1 + \beta _{24}\eta _1\xi _2 + \gamma _{21}\xi _1 \\&\quad + \gamma _{22}\xi _2 + \gamma _{23}\xi _1^2 + \gamma _{24}\xi _1\xi _2 + \gamma _{25}\xi _2^2 + \zeta _2, \end{aligned}$$

where each of the latent variables $\xi _1, \xi _2, \eta _1,$ and $\eta _2$ is measured with noise, here each is measured by three indicators (see Fig. 1).

It is important to note that this model only falls into the model class of QISEM if we disallow interactions involving $\eta _1$ simultaneously with nonlinear effects of $\xi _1$ or $\xi _2$ on $\eta _1$ in the full model. Such a model would contain terms of order 3 (e.g., $\xi _1^3$ or $\xi _1^2\xi _2$), which are not permitted in QISEM. Therefore, if any of the parameters $\beta _{22}$, $\beta _{23}$, or $\beta _{24}$ is expected to be non-zero, then $\gamma _{13}=\gamma _{14}=\gamma _{15}=0$, and vice versa.

In the following, we investigate power estimation in both linear SEM and QISEM. We choose these models because analytical estimation procedures for power exist for linear SEM under normality (see MacCallum, Browne, & Sugawara, 1996; Satorra & Saris, 1985). This analytical solution serves as a reference point. However, in the case of linear SEM under distributional misspecification, there is no general solution to estimate power other than ad-hoc sample size selections and estimating power through the relative frequency of significant effects. Therefore, we assess the performance of power estimation using MSPE for linear SEM under distributional misspecification.

Additionally, we utilize the unconstrained product indicator approach (UPI, Kelava & Brandt, 2009, Marsh et al., 2004) to estimate an interaction effect in QISEM. The UPI approach is a limited information approach fitted with the ML-fitting function for linear SEM, making it misspecified even under normality, and no power estimation procedure exists for it.

In MSPE, a probit model is fitted, and the uncertainty in parameter estimates ($AVAR(\hat{\beta }_\text {probit})$) within the probit regression fit is used to derive the sample size corresponding to the lower bound of the predicted power $\hat{N}_{\alpha ,\text {lb}}$, ensuring a power rate of $\rho$ with a type I error rate of $\alpha _\rho /2$. Therefore, the performance of the method depends on the chosen sample sizes within the fitting process. Sample sizes resulting in higher power rates exceeding $\rho >.99$ yield much higher expected uncertainty than sample sizes resulting from $\rho \le .99$. Since power rates exceeding .99 are rarely relevant in practice, we constrain the evaluation of the MSPE to desired power rates below or equal to .99 ($\rho \le .99$).

Demidenko (2007) argued that it is important to compute power specifically for the test being used. It is well known that the two-sided *z*-test is equivalent to the one-parameter Wald test, and the Wald test is asymptotically equivalent to the likelihood ratio test (LRT) (see, e.g., Buse, 1982; Engle, 1984). However, for small samples, these tests may yield different significance decisions, resulting in differing power rates (Buse, 1982; Engle, 1984). For an illustration of this discrepancy and further information see Fig. 9 in Appendix D. Further, analytical asymptotic power rates may differ from empirically estimated power rates for small *n* (as evidenced by non-overlapping confidence bands in Fig. 9 in Appendix D). This is why we use empirically derived power rates with large replication counts ($R=10^6$ per *n*) per method throughout this paper as a reference for evaluation.

**Table 1:** Power modeling methods used in simulation studies

Method/Link	Predictor	Expectation	Abbreviation
Probit	$\sqrt{n}$	unbiased	*Probit-sqrt*
Probit for two-sided test	$\sqrt{n}$	unbiased	*Wald*
Probit	*n*	biased	*Probit*
Logit	$\sqrt{n}$	biased	*Logit-sqrt*
Logit	*n*	biased	*Logit*
Linear interpolation	*n*	biased	*Naïve*

*Note.* The unbiased expectation of *Probit-sqrt* and *Wald* only refers to the one-sided and two-sided *z*-test. For the alternative methods, bias at least in intervals for the sample sizes are expected

### Overview of simulation studies and evaluation criteria

In the following two simulation studies, we evaluate the one-sided *z*-test, the two-sided *z*-test, and the LRT for linear SEM and QISEM. The proposed MSPE is applied to each of the three significance tests for each of the two models, using either a probit regression or a version of a probit regression tailored for the two-sided *z*-test (hereafter referred to as the Wald test), which is equivalent to the one-parameter Wald test (see Appendix B). Both probit regression methods use $\sqrt{n}$ as a predictor.

To assess the usefulness of the MSPE, we compared it to modeling the significance decision using a probit regression model with *n* as a predictor. Additionally, we altered the link function and added a logistic regression to model statistical power (in line with Schoemann et al., 2014), using *n* and $\sqrt{n}$ as predictors. Finally, we included a naïve approach that uses linear interpolation among four selected sample sizes with $R_j=R/4$, where $j=1,2,3,4$.

All approaches were inverted to solve for the required sample size $\hat{N}_\alpha$. Furthermore, the uncertainty of the estimation process was modeled by using the lower bound of the predicted interval for each power value to estimate $\hat{N}_{\alpha ,\text {lb}}$. For the naïve approach, the normal theory confidence interval was computed for each sample size, and the lower bounds of these four confidence intervals were linearly interpolated to estimate $\hat{N}_{\alpha ,\text {lb}}$. For the two logistic regression approaches $\hat{N}_{\alpha ,\text {lb}}$ results as

$$\begin{aligned} \hat{N}_{\alpha ,\text {lb},\text {logit-sqrt}}&\approx \left\lceil \left( \frac{\log \left( \frac{\hat{\rho }_{\hat{\rho }_\text {lb} = \rho }}{1-\hat{\rho }_{\hat{\rho }_\text {lb} = \rho }}\right) -\hat{\beta }_{0,\text {logit-sqrt}}}{\hat{\beta }_{1,\text {logit-sqrt}}}\right) ^2 \right\rceil ,\\ \hat{N}_{\alpha ,\text {lb},\text {logit}}&\approx \left\lceil \frac{\log \left( \frac{\hat{\rho }_{\hat{\rho }_\text {lb} = \rho }}{1-\hat{\rho }_{\hat{\rho }_\text {lb} = \rho }}\right) -\hat{\beta }_{0,\text {logit}}}{\hat{\beta }_{1,\text {logit}}} \right\rceil , \end{aligned}$$

where $\hat{\beta }_{0,\text {logit-sqrt}}$ and $\hat{\beta }_{1,\text {logit-sqrt}}$, and $\hat{\beta }_{0,\text {logit}}$ and $\hat{\beta }_{1,\text {logit}}$ represent the estimated coefficients of the logit regression with $\sqrt{n}$ and *n* as predictors, respectively. Additionally, $\hat{\rho }_{\hat{\rho }_\text {lb}=\rho }$ is the power rate ensuring that the corresponding lower bound $\hat{\rho }_\text {lb}$ is equal to the desired power rate $\rho$. This value can be expected to be different for the two logistic regression models.

Since the methods of the MSPE are theoretically derived and correspond asymptotically to the ML estimate of power, they should be unbiased. For an overview of the evaluated power estimation methods, please refer to Table 1 and their respective abbreviations.

All analyses in the following were conducted in R (R Core Team, 2023). In order to evaluate the performance of the MSPE and the related methods, we simulated $10^6$ replications for each reference sample sizes per model class. Due to the high computational cost, we restricted the number of empirical power rates used as references to 31 and 22 for SEM and QISEM, respectively, as $10^6$ data sets per *N* had to be simulated and fit running the methods in order to achieve adequate precision. For linear SEM reference sample sizes were spaced equally between 50 and 650 ($N = 50, 70, 90,$ $\dots , 630, 650$), while for QISEM these reference sample sizes were spaced equally between 150 and 1200 ($N = 150, 200,$ $\dots , 1150, 200$). We evaluated the empirical power by the relative frequency of a significant test after removing all non-converged samples. See Fig. 10 in Appendix D for rates of proper solutions.

**Fig. 2:**
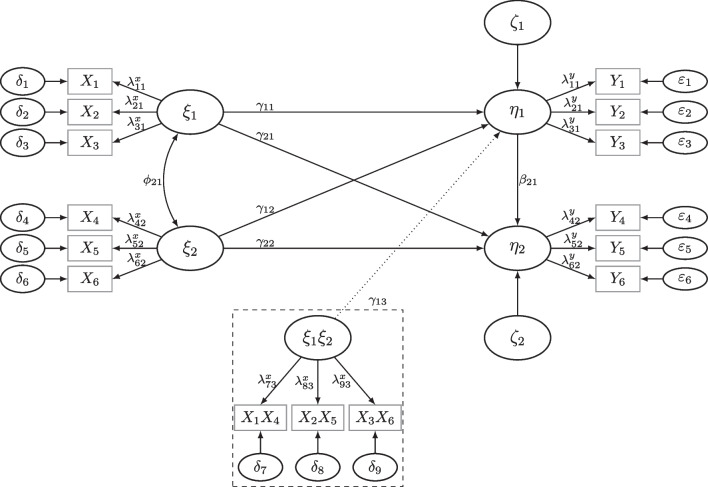
Linear SEM and QISEM as used in Simulation Study 1 and 2. *Note*. Model in *solid lines* refers to linear SEM studied in Simulation Study 1. The *dotted line* indicates moderation effect of $\xi _1\xi _2$ on $\eta _1$, which is only included in the QISEM studied in Simulation Study 2. The *dashed box *shows product indicators formed within the unconstrained product indicator approach used to fit the QISEM

In the two simulation studies, we altered the number of total replications *R* and the distributional specification (correctly specified and misspecifed) for each model class to study the MSPE. In total, we simulated $10^3$ repetitions for each of the conditions per method and references were computed for all combinations of conditions. Three evaluation criteria for the performance of the MSPE were used: First, the bias in estimated sample size $\hat{N}_\alpha$ and $\hat{\rho }$ and second, the root mean squared error (RMSE) were calculated across all $10^3$ replications and averaged across the reference sample sizes and empirical power rates. Third, we used the probability of the lower bound $\hat{N}_{\alpha ,\text {lb}}$ to be smaller than the reference sample size, which serves as a proxy of the type I error rate:

$$\begin{aligned} \text {Bias in }N_\alpha&= \frac{1}{\text {Reps}_c}\sum _{i=1}^{\text {Reps}_c} \hat{N}_{\alpha ,\star ,i} - N_\alpha \\ \text {Bias in }\rho \text { for } N_\alpha&= \frac{1}{\text {Reps}_c}\sum _{i=1}^{\text {Reps}_c} \hat{\rho }_{N_\alpha ,i} - \hat{\rho }_{\text {ref},N_\alpha }, \\ \text {RMSE in }N_\alpha&= \sqrt{\frac{1}{\text {Reps}_c}\sum _{i=1}^{\text {Reps}_c} \left( \hat{N}_{\alpha ,\star ,i} - N_\alpha \right) ^2},\\ \text {Type I error}&=\frac{1}{\text {Reps}_c}\sum _{i=1}^{\text {Reps}_c} \textbf{1}_{\{\hat{N}_{\alpha ,\star ,i} < N_\alpha \}}, \end{aligned}$$

where the number of converged samples is denoted by Reps$_c$ which should be close to $10^3$, $\hat{N}_{\alpha ,\star ,i}$ is the computed required sample size for replication *i* using the different methods based on the computed power rate $\hat{\rho }_{\text {ref},N_\alpha }$, $\hat{\rho }_{\text {ref},N_\alpha }$ is the relative frequency of a significant test representing the power rate for sample size $N_\alpha$, and $\hat{\rho }_{N_\alpha ,i}$ is the computed power rate for $N_\alpha$ for replication *i* using the different methods to model power. $\textbf{1}_{\{\hat{N}_{\alpha ,\star ,i} < N_\alpha \}}$ is the indicator function for the that the predicted sample sizes considering sample variability is smaller than the true required sample size denoted by ${\{\hat{N}_{\alpha ,\star ,i} < N_\alpha \}}$. The type I error rate is expected to be close to $\alpha _\rho /2 = 2.5\%$ for the MSPE. The performance of the MSPE methods, *Probit-sqrt* and *Wald*, are compared to alternative approaches (see Table 1).

Finally, we computed the type I error rate for each used statistical test (one-sided and two-sided *z*-test and the LRT) for three selected sample (small, medium, large).

### Simulation study 1: Application of MSPE to linear SEM

Simulation Study 1 considers a linear SEM with two exogenous and two endogenous latent variables in the structural model (see Fig. 2). This model is extended to a moderated mediation model in Simulation Study 2. The structural model of the linear SEM is given by

$$\begin{aligned} \eta _1&= .2\xi _1 + .5\xi _2 + \zeta _1,\\ \eta _2&= .3\eta _1 + .4\xi _1 + .3\xi _2 + \zeta _2. \end{aligned}$$

Our goal was to estimate the power for a single regression parameter within the structural model, i.e., the effect $\xi _1\rightarrow \eta _1$ denoted by $\gamma _{11} (=.2)$ in Fig. 2. For our simulation, we set the variance of $\zeta _1$ and $\zeta _2$ so that all latent variables ($\xi _1,\xi _2,\eta _1,\eta _2$) were standardized. All latent variables were measured by three indicator variables. Measures of reliability, such as internal consistency (estimated using McDonald’s $\omega$, McDonald, 1999) or maximal reliability (estimated using coefficient *H*, Hancock & Mueller, 2001) were rather high ($\Lambda =[1,.8,.7]', \omega =.85, H =.88$).

**Fig. 3:**
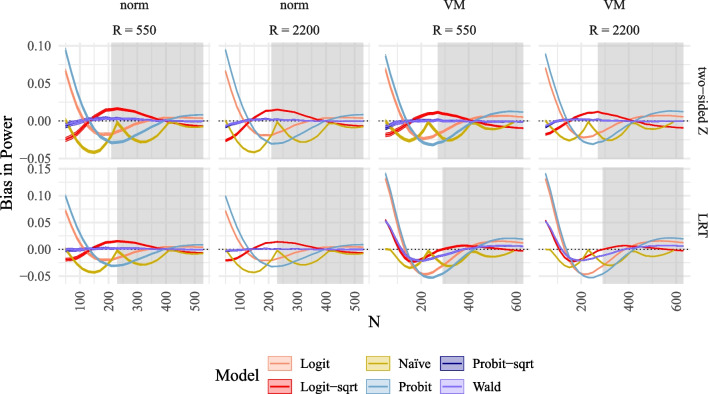
Bias of MSPE in power plotted against true *N* compared to alternative methods to model power for SEM. *Note*. Bias of MSPE in power plotted against true *N* compared to alternative methods to model power for SEM for the different power models (logit, logit with $\sqrt{n}$ as predictor, probit, probit with $\sqrt{n}$ as predictor, naïve with 4 *n*s, Wald with $\sqrt{n}$ as predictor) for two tests in rows (two-sided *z*-test, LRT), and for two numbers of replications *R* and two different distributions in columns (norm = normal $\xi$, VM = Vale-Maurelli $\xi$). *Shaded areas* correspond to 95% confidence bands. The *grey background* corresponds to reference power rates exceeding .70

In total, we simulated $10^3$ repetitions for the MSPE and the other parametric approaches using either $R=550, 1100$, or 2200 data sets and corresponding significance decision to fit the power models uniformly distributed on $N=51,\dots ,600$. This means that for $R=550$, each sample size ($51,\dots ,600$) is used once, whereas for $R=2200$, each sample size is used four times. For the naïve approach, the number of replications *R* were divided by 4 (rounded to the next integer) and sample sizes were equally spaced between 50 and 599. Data were simulated under normality (utilizing the mvtnorm package, Genz & Bretz, 2009) or under non-normality using the Vale and Maurelli (1983, VM) approach with little to moderate skewness ranging from .7 to 2.3 and kurtosis ranging from 2.5 to 7.2 for the manifest variables (utilizing the semTools package, Jorgensen, Pornprasertmanit, Schoemann, & Rosseel 2020). Skewness and kurtosis values in this interval have been used in several other simulation studies (e.g., Brandt et al., 2014; Flora & Curran, 2004; Rhemtulla, Brosseau-Liard, & Savalei, 2012). We acknowledge that the VM approach for simulating non-normal data is not the most general method due to its normal copula (Foldnes & Grønneberg, 2015). However, because it has been frequently used in previous simulation studies, we included it in our study. Nonetheless, the proposed MSPE should be applicable to all types of non-normal data that allow for unbiased estimates and (asymptotically) normal parameter estimates, i.e., a well-behaved *z*-test. SEM were fitted using ML estimation for normal data and the robust variant (MLR) for non-normal data using lavaan (Rosseel, 2012). We used the Satorra-Bentler robust variant of the likelihood ratio test (Satorra & Bentler, 2010) within the MSPE and related methods as well as in the computation of the reference power rates (see Appendix C for more details).

Since the results for the one-sided *z*-test were nearly identical to those of the two-sided *z*-test, we have omitted the one-sided *z*-test results from the following analysis. Additionally, we excluded the $R=1100$ condition, as it did not provide any insights beyond those obtained from the $R=550$ and $R=2200$ conditions. All conditions are illustrated in the additional plots available in the OSF repository.

#### Bias and RMSE

Figure 3 illustrates the bias in the estimated power across different sample sizes. It is evident that only the methods of the MSPE (*Probit-sqrt* and *Wald*) are unbiased across all sample sizes for both the one-sided and two-sided *z*-tests. They also exhibit small bias for the LRT under normality but significant bias for the LRT under non-normality. In contrast, all alternative methods used to model power demonstrate substantial intervals of sample sizes with bias. Although confidence intervals decrease in size with increasing *R*, the mean bias remains unaffected by *R*.

**Fig. 4:**
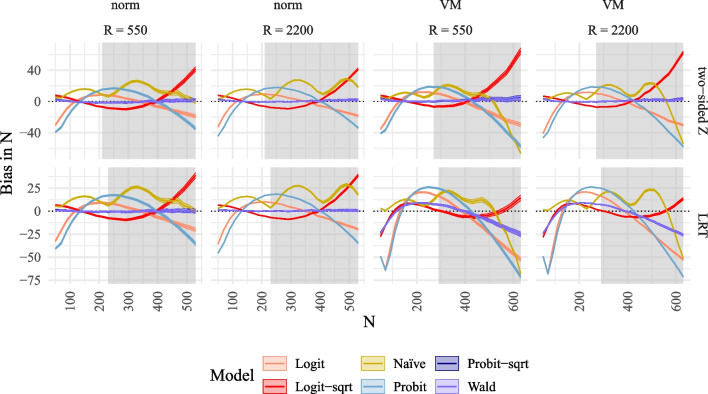
Bias of MSPE in $\hat{N}_\alpha$ plotted against true *N* compared to alternative methods to model power for SEM. *Note*. Bias of MSPE in $\hat{N}_\alpha$ plotted Against true *N* compared to alternative methods to model power for SEM for the different power models (logit, logit with $\sqrt{n}$ as predictor, probit, probit with $\sqrt{n}$ as predictor, naïve with 4 *n*s, Wald with $\sqrt{n}$ as predictor) for two tests in rows (two-sided *z*-test, LRT), and for two numbers of replications *R* and two different distributions in columns (norm = normal $\xi$, VM = Vale-Maurelli $\xi$). The *shaded areas* correspond to 95% confidence bands. The *grey background* corresponds to reference power rates exceeding .70

**Fig. 5:**
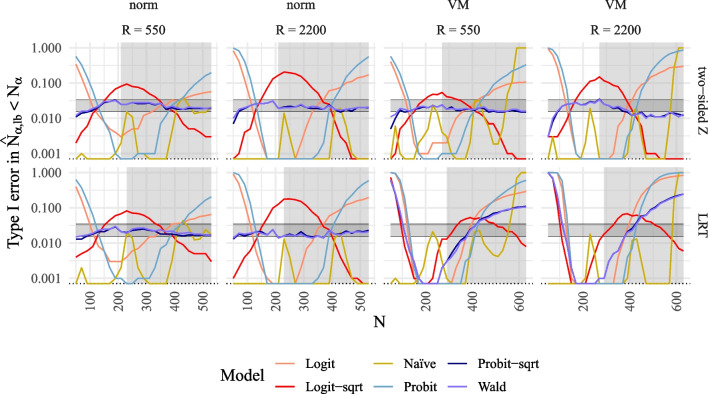
Type I error rate for the MSPE compared to alternative methods to model power for SEM. *Note*. Type I error rates of MSPE in $\log _{10}$-scale plotted against true *N* compared to alternative methods to model power for SEM for the different power models (logit, logit with $\sqrt{n}$ as predictor, probit, probit with $\sqrt{n}$ as predictor, naïve with 4 *n*s, Wald with $\sqrt{n}$ as predictor) for two tests in rows (two-sided *z*-test, LRT), and for two numbers of replications *R* and two different distributions in columns (norm = normal $\xi$, VM = Vale-Maurelli $\xi$). The* grey background* corresponds to reference power rates exceeding .70. The *shaded horizontal areas* correspond to 95% confidence bands around the expected 2.5% value

The unbiasedness observed in power rates translates into unbiasedness in predicted sample sizes, as shown in Fig. 4. The results indicate that the methods of the MSPE (*Probit-sqrt* and *Wald*) predict unbiased sample sizes for both one-sided and two-sided *z*-tests, as well as for the LRT under normality. In contrast, all alternative methods exhibit regions of sample sizes with substantial bias. While confidence intervals decrease in size with increasing *R*, the average bias does not decrease with increasing *R* (see also Fig. 11 in Appendix D). Similarly, the RMSE decreases with increasing *R*, but only for the methods of the MSPE do the RMSE values approach zero (see Fig. 12 in Appendix D). Numerical values for selected sample sizes under the condition $R=2000$ are provided in Tables 4, 5, and 6 in Appendix E. These values indicate that higher required sample sizes are obtained for non-normal data. Furthermore, Table 2 in Appendix E presents the type I error rates for selected sample sizes, revealing slight inflation of type I error rates with maximum values of .063. However, with increasing sample size, the type I error rates per statistical test approach the required value of 5%.

#### Type I error rate

Figure 5 illustrates the proportion of the predicted sample size $\hat{N}_{\alpha ,\text {lb}}$ corresponding to the lower bound of predicted power that is smaller than $N_\alpha$, which serves as a type I error rate. The results indicate that the methods of the MSPE (*Probit-sqrt* and *Wald*) demonstrate acceptable type I error rates across all sample sizes for both one-sided and two-sided *z*-tests, as well as for the LRT under normal data conditions. However, type I error rates are inflated or reduced for the LRT under non-normal data conditions. Conversely, all alternative methods for modeling power exhibit substantial inflation or reduction in type I error rates for some sample sizes.

To summarize, the methods of the MSPE (*Probit-sqrt* and *Wald*) show good performance in terms of bias, RMSE, and type I error rates across all conditions for the one-sided and two-sided *z*-test, as well as the LRT under normality, while all alternative methods to model power show substantial bias and inflated or reduced type I error rates. Consequently, only the MSPE succeeds in predicting power and corresponding sample size.

### Simulation study 2: Application of MSPE to QISEM

In Simulation Study 2, we extend the linear SEM of Simulation Study 1 by an interaction/moderation effect between $\xi _1$ and $\xi _2$ on $\eta _1;\ \xi _1\xi _2\rightarrow \eta _1$ (see also Fig. 2). The structural model is given by

$$\begin{aligned} \eta _1&= .2\xi _1 + .5\xi _2 + .1\xi _1\xi _2 + \zeta _1,\\ \eta _2&= .3\eta _1 + .4\xi _1 + .3\xi _2 + \zeta _2. \end{aligned}$$

The goal of this study was to estimate the power for the interaction effect $\xi _1\xi _2\rightarrow \eta _1$ denoted by $\gamma _{13} (=.1)$ in Fig. 2. Measurement models were set as in Simulation Study 1, residual variances were adapted to yield standardized latent variables $\xi _1,\xi _2,\eta _1,$ and $\eta _2$. To fit the models, we employed the (unconstrained) product indicator approach (Kelava & Brandt, 2009; Marsh et al., 2004), using matched product indicators and the double mean centering approach (Lin, Wen, Marsh, & Lin, 2010), computed with semTools (Jorgensen et al., 2020), and MLR estimation in lavaan (Rosseel, 2012).

Within this study, we introduced an interaction effect and computed product indicators, extending the previous simulation study. Additionally, in contrast to the previous section where ML estimation was used for the SEM under correctly specified models, MLR estimation was employed here. This decision was made due to the introduction of non-normality into the model by the nonlinear effect, rendering ML estimation using the ML-fitting function inappropriate (e.g., Jöreskog & Yang, 1996).

For assessing power in more complex QISEM scenarios, including multiple moderation effects, recent work by Irmer, Klein, and Schermelleh-Engel (2024) offers valuable insights. They provide R implementations in the powerNLSEM package (Irmer, 2024). Additionally, the Satorra-Bentler robust variant of the likelihood ratio test (Satorra & Bentler, 2010) was utilized within the MSPE and related methods, as well as in the computation of reference power rates. Further details are available in Appendix C.

Similarly to Simulation Study 1, we used $10^3$ repetitions for $R=550, 1100, 2200$ for the MSPE and the other parametric methods spread equally across sample sizes $N=140, 142, 144,$ $\dots , 1236, 1238$, while for the naïve approach *R* was divided by 4 and sample sizes were spread equally between 140 and 1241 for the naïve approach. All variables were simulated to be normal, except for the dependent variables which were model-implied non-normal due to the interaction effect. Comparable to the linear SEM, we added a non-normal condition in which the latent predictors $\xi _1,\xi _2$ were simulated to be skewed using the Vale and Maurelli (1983) approach with skewness 1.7, 2.3 and kurtosis 7.2, 6.5, respectively. These values represent medium skewness and they are close to typically used values in simulation studies of skewness 2 and kurtosis 7 (see, e.g., Brandt et al., 2014). Again, we acknowledge that the VM approach is not the most general method for generating non-normal data. Future research could explore more comprehensive procedures.

**Fig. 6:**
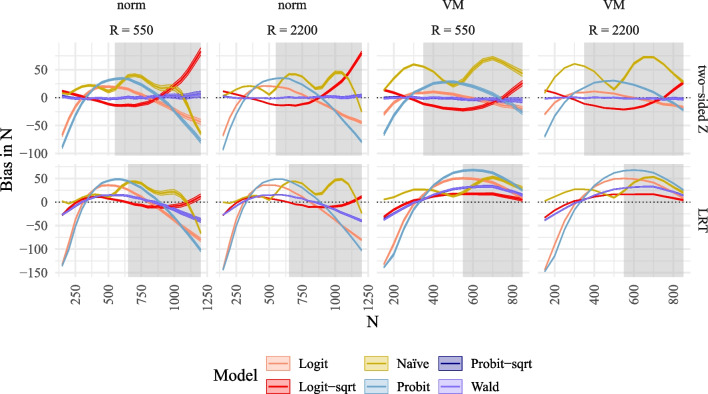
Bias of MSPE in $\hat{N}_\alpha$ plotted against true *N* compared to alternative methods to model power for QISEM. *Note*. Bias of MSPE in $\hat{N}_\alpha$ plotted against true *N* for QISEM for the different power models (logit, logit with $\sqrt{n}$ as predictor, probit, probit with $\sqrt{n}$ as predictor, naïve with 4 *n*s, Wald with $\sqrt{n}$ as predictor) for three tests in rows (one-sided, two-sided, LRT), and for two tests in rows (two-sided *z*-test, LRT), and for two numbers of replications *R* and two different distributions in columns (norm = normal $\xi$, VM = Vale-Maurelli $\xi$). The *shaded areas* correspond to 95% confidence bands. The *grey background* corresponds to reference power rates exceeding .70

#### Bias and RMSE

Figure 6 displays the bias in predicted sample size plotted against the reference sample sizes for the MSPE and alternative power prediction methods. Results indicate minimal to no bias for the one-sided and two-sided *z*-tests when employing the MSPE (*Probit-sqrt* and *Wald*). Conversely, significant bias is observed across all conditions for some sample sizes when using alternative power prediction methods. Notably, all methods exhibit substantial bias in predicted sample size for the LRT. Although variability decreases with increasing *R*, bias remains unaffected by *R* (refer to Figs. 13, 14, and 15 in Appendix D for further details).

Numerical values for selected sample sizes under the condition $R=2000$ are provided in Tables 7, 8, and 9 in Appendix E. Unlike linear SEM, these values suggest that higher required sample sizes are necessary for normal data in QISEM scenarios. Additionally, Table 3 in Appendix E presents type I error rates per test for selected sample sizes. These indicate a slight inflation of type I error rates, with maximum values of .072 and .081 for the one-sided and two-sided tests, respectively. Conversely, type I error rates for the LRT were slightly lower than expected. However, as sample size increased, type I error rates per statistical test approached the required value of 5%.

#### Type I error rate

**Fig. 7:**
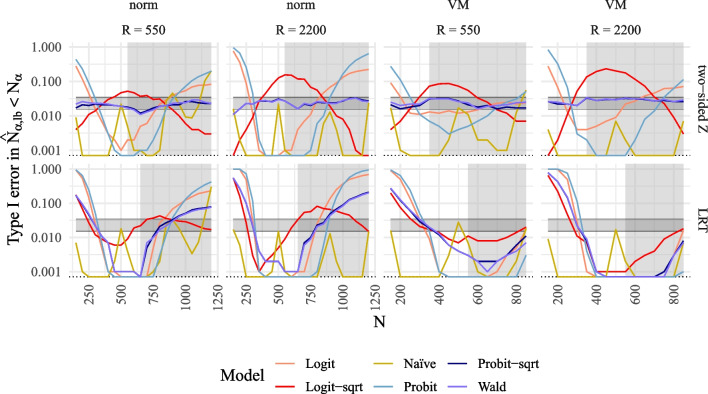
Type I error rate for the MSPE compared to alternative methods to model power for QISEM. *Note*. Type I error rates of MSPE in $\log _{10}$-scale plotted against true *N* compared to alternative methods to model power for QISEM for the different power models (logit, logit with $\sqrt{n}$ as predictor, probit, probit with $\sqrt{n}$ as predictor, naïve with 4 *n*s, Wald with $\sqrt{n}$ as predictor) for two tests in rows (two-sided *z*-test, LRT), and for two numbers of replications *R* and two different distributions in columns (norm = normal $\xi$, VM = Vale-Maurelli $\xi$). The *grey background* corresponds to reference power rates exceeding .70. *Shaded horizontal areas* correspond to 95% confidence bands around the expected 2.5% value

**Fig. 8:**
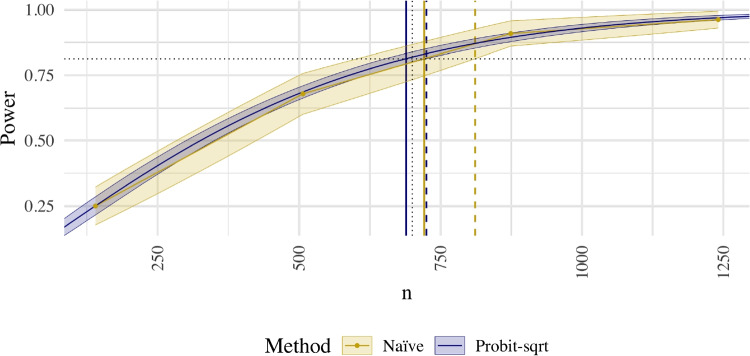
Example application of the MSPE in comparison to linear interpolation of power for the QISEM used in Simulation Study 2. *Note*. Predicted power for $R=2200$ replications for the QISEM for $n=140, 507, 874, 1241$ (550 replications each), as studied in simulation study 2 for the MSPE (Probit-sqrt) in comparison to linear interpolation of power rates (naïve). *Shaded areas* correspond to 95% confidence intervals. *Vertical lines *indicate $\hat{N}_\alpha$, *vertical dashed lines* indicate $\hat{N}_{\text {lb},\alpha }$, and *vertical dotted line* indicates $N_\alpha =700$ as reference. All required sample sizes belong to $\rho \approx .812$, which is the simulated power rate for $n=700$ based on $10^6$ simulated data sets. The sample sizes for the MSPE were $\hat{N}_{\alpha , \text {MSPE}} = 689$, $\hat{N}_{\text {lb}, \alpha , \text {MSPE}} = 725$, and for linear interpolation they were $\hat{N}_{\alpha , }$_naïve_ $= 721$, $\hat{N}_{\text {lb}, \alpha , }$_naïve_ $= 811$

The type I error rates for the methods of the MSPE (*Probit-sqrt* and *Wald*) are mostly within the acceptable range for both one-sided and two-sided *z*-tests. Conversely, they are largely inflated or too small for some regions of reference sample sizes in the case of the LRT. On the other hand, alternative methods exhibit inflated or reduced type I error rates for some regions in all tests and distributional conditions (refer to Fig. 7).

In summary, the findings of Simulation Study 1 are replicated for QISEM, where the methods of the MSPE (*Probit-sqrt* and *Wald*) exhibited favorable performance in terms of bias, RMSE, and type I error rates across all conditions for both the one-sided and two-sided *z*-tests. Additionally, they demonstrated minimal bias for the LRT under normality. Conversely, all alternative methods displayed significant bias and inflated or reduced type I error rates. Consequently, once again, only the MSPE proved effective in predicting power and the corresponding required sample size.

## Discussion

Closed-form power estimation is unavailable for many model classes. In this paper, we proposed the new model-implied simulation-based power estimation (MSPE) method applicable to a wide range of estimators and models, including models with misspecifications. The MSPE utilizes the distribution of parameter estimates and models their significance decision using the one-sided or two-sided *z*-test by a probit regression model with the square root of the sample size as a predictor. The probit regression model is then inverted to solve for the required sample size. Since the MSPE is a simulation-based procedure, the power estimates are subject to sample variability. This aspect is included in the procedure for an optimized calculation of the required sample size by using the confidence band around the predicted power rates resulting from the probit regression approach in the inversion process. For the two-sided *z*-test, which is equivalent to the one-parameter Wald test, we proposed a variation of the probit regression that results from the absolute value used in the significance decision. This version of the probit regression can also be inverted to find the required sample size for a predefined power rate. The important difference between our approach and conventional power analysis lies in the idea to fit a parametric model for the power curve, as opposed to simple point-wise power analysis for selected sample sizes. By setting up a parametric model, correlational information between power rates and sample sizes is used for an unbiased and efficient calculation of expected power rates. As a consequence, this yields narrower confidence intervals, because additional statistical information is used.

We illustrated the performance of the MSPE for sub-models of nonlinear SEM: linear SEM and quadratic and interaction SEM (QISEM) for both the one-sided and two-sided *z*-test. We compared the results to the modeling of the significance decision using the likelihood ratio test (LRT). In two simulation studies, we studied correctly specified models and models with distributional misspecifications by comparing the performance of the MSPE to an empirically estimated reference. Results suggest that the MSPE is an unbiased method to estimate power and required sample size with acceptable type I error rates for the one-sided and two-sided *z*-test. All alternative methods to model power, such as logistic regression with sample size as a predictor (as e.g., used in Schoemann et al., 2014) or the naïve approach using an ad-hoc selection of sample sizes with linear interpolation between the selected points showed poor performance with regard to bias and type I error rates in comparison to the MSPE, at least for regions of sample sizes. The MSPE also worked well for the LRT in some conditions, which is most probably due to the asymptotic equivalence of the LRT and the two-sided *z*-test (Engle, 1984) under ideal circumstances. For non-normal data, corrections for the LRT were applied (Satorra & Bentler, 1994). However, the relationship between power and sample size was not adequately modeled by a probit regression with $\sqrt{n}$ as the predictor under non-normality, indicating that a different link function may be necessary in these cases. This may be because not all corrections for LRT (or $\chi ^2$ tests) are inherently consistent under non-normality (Foldnes & Olsson, 2015; Foldnes & Grønneberg, 2018; Foldnes, Moss, & Grønneberg, 2024). Only the distribution-free estimator proposed by Browne (1984) is consistent under general non-normality. However, it requires extremely large sample sizes (Foldnes et al., 2024), making it impractical for most applied research and power analyses.

Hence, these results suggest that taking the distribution of parameter estimates and, therefore, the distribution of significance decisions directly into account, benefits the accuracy in predicting power using simulation methods. The probit regression outperforms the logit regression as the significance decision results from the *z*-value which follows a normal distribution with unit variance. This normality is modeled by the probit regression. Further, precision of estimates is related to the factor of the inverse of root-*n* to variability of parameter estimates. The standard deviation of parameter estimates decreases anti-proportional to the square root of the sample size. This standard deviation is then used in the computation of the *z*-value. Therefore, the square root of the sample sizes as predictor in the probit regression naturally results as a consequence of the derivation of the *z*-value.

**Fig. 9:**
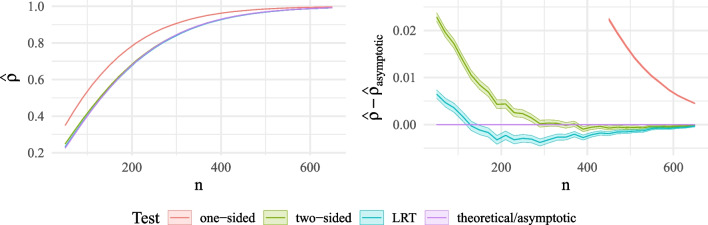
Empirical power and analytical asymptotic power plotted against *n*. *Note*. Empirical power and analytical asymptotic power plotted against *n* for the one-sided and two-sided *z*-test and the LRT for the linear SEM for normally distributed data used in Simulation Study 1 based on $R=10^6$ replications per *n*. On the right-hand side, the one-sided *z*-test is cutoff for some values for clarity as only the two-sided *z*-test (Wald test) and the LRT should be asymptotically equivalent; the difference to the analytical asymptotic power is depicted. The *shaded area* corresponds to 95% confidence bands

**Fig. 10:**
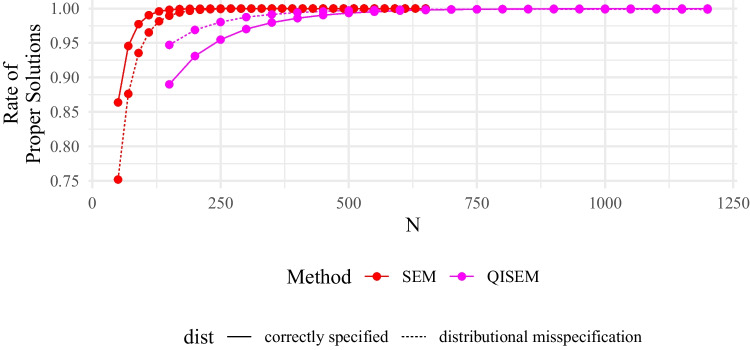
Rate of proper solutions in simulation studies. *Note*. Rate of proper solutions that can be used to fit MSPE per method and distributional condition within the $10^6$ fitted models per *N*

**Fig. 11:**
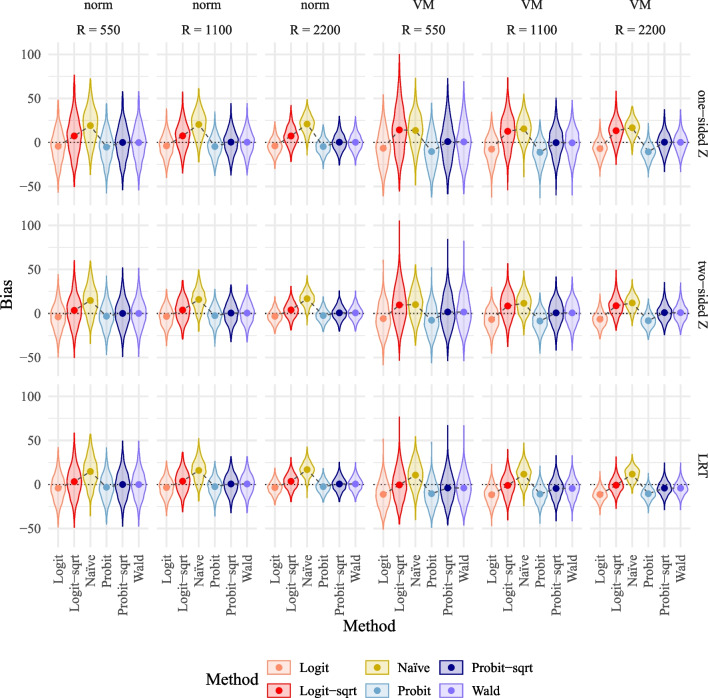
Mean bias of MSPE in $\hat{N}_\alpha$ compared to alternative methods to model power for SEM. *Note*. Mean bias of MSPE in $\hat{N}_\alpha$ for SEM for each replication of the different power models (logit, logit with $\sqrt{n}$ as predictor, probit, probit with $\sqrt{n}$ as predictor, naïve with 4 *n*s, Wald with $\sqrt{n}$ as predictor) for three tests in rows (one-sided, two-sided, LRT), and for three numbers of replications *R* and two different distributions in columns (norm = normal $\xi$, VM = Vale-Maurelli $\xi$). *Points connected by lines *represent means across all replications

**Fig. 12:**
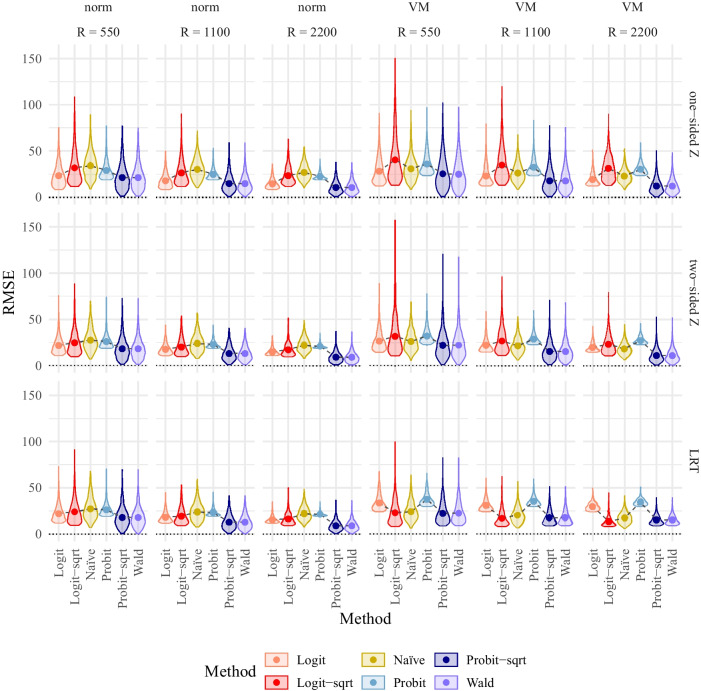
RMSE of MSPE in $\hat{N}_\alpha$ compared to alternative methods to model power for SEM. *Note*. RMSE of MSPE in $\hat{N}_\alpha$ for SEM for each replication of the different power models (logit, logit with $\sqrt{n}$ as predictor, probit, probit with $\sqrt{n}$ as predictor, naïve with 4 *n*s, Wald with $\sqrt{n}$ as predictor) for three tests in rows (one-sided, two-sided, LRT), and for three numbers of replications *R* and two different distributions in columns (norm = normal $\xi$, VM = Vale-Maurelli $\xi$). *Points connected by lines* represent means across all replications

**Fig. 13:**
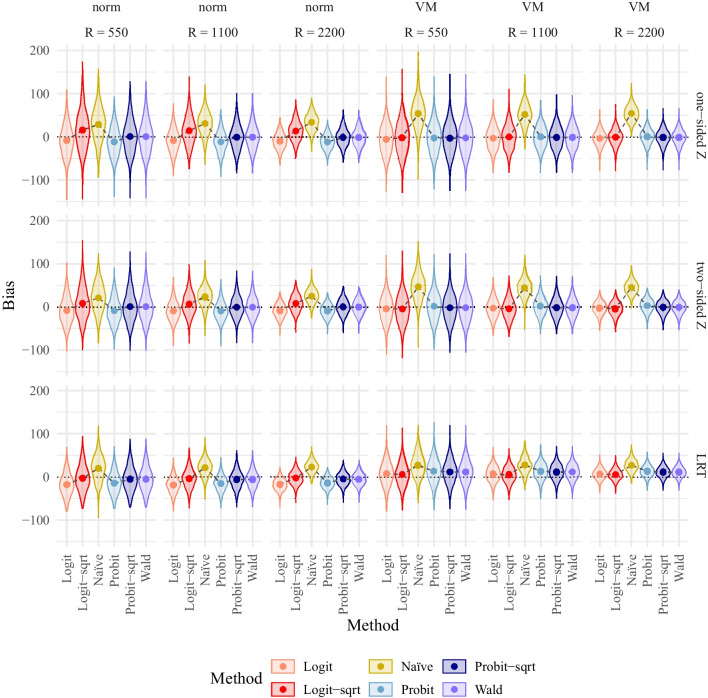
Mean bias of MSPE in $\hat{N}_\alpha$ compared to alternative methods to model power for QISEM. *Note*. Mean bias of MSPE in $\hat{N}_\alpha$ for QISEM for each replication of the different power models (logit, logit with $\sqrt{n}$ as predictor, probit, probit with $\sqrt{n}$ as predictor, naïve with 4 *n*s, Wald with $\sqrt{n}$ as predictor) for three tests in rows (one-sided, two-sided, LRT), and for three numbers of replications *R* and two different distributions in columns (norm = normal $\xi$, VM = Vale-Maurelli $\xi$). Points connected by lines represent means across all replications

**Fig. 14:**
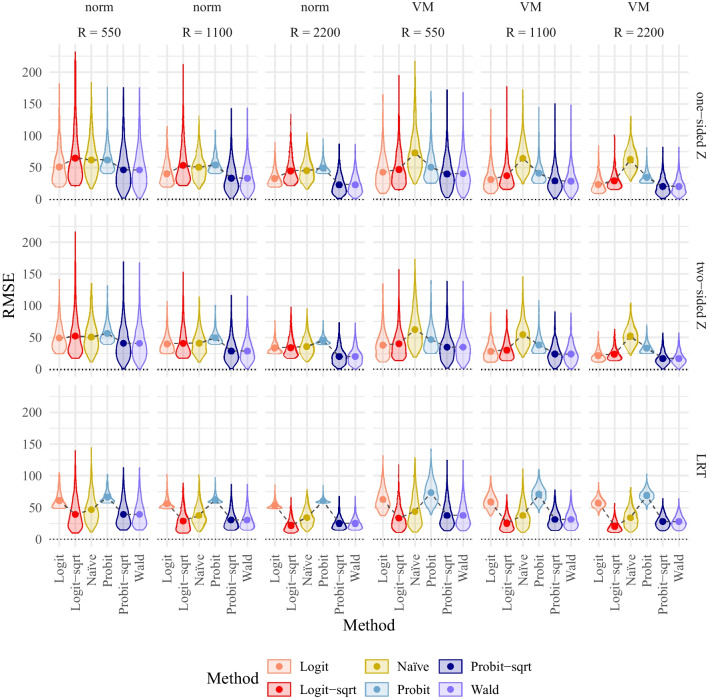
RMSE of MSPE in $\hat{N}_\alpha$ for QISEM. *Note*. RMSE of MSPE in $\hat{N}_\alpha$ for QISEM for each replication of the different power models (logit, logit with $\sqrt{n}$ as predictor, probit, probit with $\sqrt{n}$ as predictor, naïve with 4 *n*s, Wald with $\sqrt{n}$ as predictor) for three tests in rows (one-sided, two-sided, LRT), and for three numbers of replications *R* and two different distributions in columns (norm = normal $\xi$, VM = Vale-Maurelli $\xi$). *Points connected by lines* represent means across all replications

**Fig. 15:**
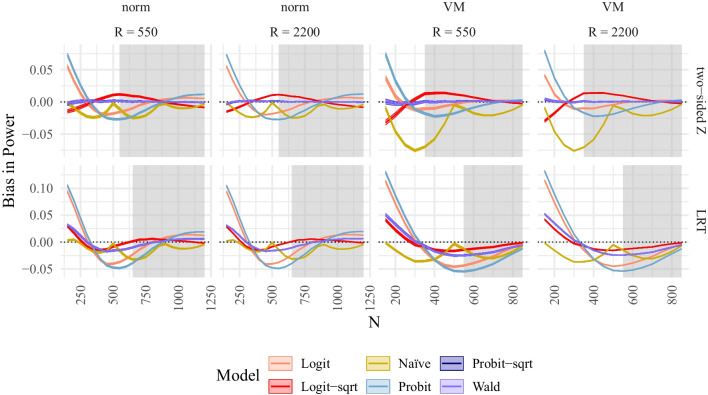
Bias of MSPE in power plotted against true *N* compared to alternative methods to model power for QISEM. *Note*. Bias of MSPE in power plotted against true *N* for QISEM for the different power models (logit, logit with $\sqrt{n}$ as predictor, probit, probit with $\sqrt{n}$ as predictor, naïve with 4 *n*s, Wald with $\sqrt{n}$ as predictor) for two tests in rows (two-sided *z*-test, LRT), and for two numbers of replications *R* and two different distributions in columns (norm = normal $\xi$, VM = Vale-Maurelli $\xi$). The *grey background *corresponds to reference power rates exceeding .70. The *shaded areas* correspond to confidence intervals

Consequently, we believe the MSPE fills a gap in estimating power for models without analytical power solutions. To date, only analytical power estimation is available for linear SEM under normality for the likelihood ratio test (MacCallum et al., 1996; Satorra & Saris, 1985), with implementations, for instance, in the semPower package (Moshagen & Bader, 2024) in R (R Core Team, 2023). However, it is important to ensure that the estimated power used in the research actually matches the significance decision used (Demidenko, 2007). If the *z*-test is to be used, the power must be estimated for this test and not for the LRT, as these tests may have different power rates in finite samples (Engle, 1984); see also Fig. 9. For linear SEM under non-normality, only simulation-based procedures are available, for instance, in the semPower package (Moshagen & Bader, 2024). Using these procedures, for a predefined sample size, power is estimated via simulation by the relative frequency of a significant effect. However, no method was yet available to solve for the required sample size for a predefined desired power rate, because only linear interpolation across ad-hoc selected sample sizes is possible or the estimation of power for every sample size, which is very time-consuming.

Up to now, for latent nonlinear models including moderation effects, power estimation procedures were only available for manifest regression. When estimating power for models with latent variables, manifest scale scores are used in a moderated regression model (Shieh, 2009). However, if scale scores are used as proxies for latent variables, they generally underestimate the effects, especially the interaction effects, due to the low reliability of the interaction term (Busemeyer & Jones, 1983). Further, the power derived from moderated regression analysis does not model the uncertainty resulting from the estimation process of scale means although it is a two-step approach (White, 1996, p. 107). The estimation of scale means in the first step results in additional variability in parameter estimates which is not modeled in power estimates used for moderated regression. Hence, using power rates derived from moderated regression will generally overestimate power and thus underestimate the required sample size.

The MSPE can be applied to all coefficients in the model simultaneously. Hence, when conducting power analyses for several coefficients, the computation time is not increased, but the minimal sample size is derived by examining the coefficient with the smallest effect size relative to its implied standard error. This is implemented, for instance, in the powerNLSEM package (Irmer, 2024), which provides an implementation to conduct power analyses for QISEM for several parameters simultaneously for different QISEM estimation methods (Irmer et al., 2024).

### Limitations and future research

Although the MSPE showed good performance in the two simulation studies for two different model classes under ideal circumstances as well as distributional misspecifications, of course, there are some limitations that are reported next and future directions are provided.

In this paper, we exclusively focused on evaluating the statistical power of individual parameters, which corresponds to a local power analysis. Future research should consider global power analysis and examine whether MSPE can be used in such scenarios.

The MSPE approach relies on the asymptotic distribution of the parameter estimates; hence, it is not an exact analytical method to model power. This implies that if the assumptions underlying the estimators are significantly different from normality within the sample sizes used in the probit regression of our proposed MSPE, the resulting power estimates might deviate from the true values. For instance, the indirect effect in mediation analysis has been shown to be non-normally distributed in small samples (Preacher & Hayes, 2008). Consequently, the method provides an approximation of power in these scenarios. Deriving required sample sizes from the predicted power rates might result in an estimation of the required sample size that yields a power rate lower than desired. Hence, it is imperative to conduct further research to analyze this issue in more detail and develop techniques to determine the reliability of the results. This might include investigating non-parametric methods for assessing the relationship between sample size and power, considering alternative functions of sample size (*n*) within parametric models beyond the square root, which could potentially refine the connection for smaller sample sizes, and exploring different probability distributions for the significance decision.

An important limitation not addressed in this article is the choice of sample sizes used to fit the MSPE. The selected sample sizes will influence the precision of the estimation of power and, consequently, the estimation of the required sample size. Hence, prior knowledge on the expected power per sample size is necessary to achieve good estimates within the MSPE without the need for extreme replication counts *R*. Only extreme replication counts *R* would allow for arbitrary choices in sample size. Future research should address this issue by examining the possibility of adaptive algorithms that can be used for the selection of sample sizes.

Finally, while the MSPE approach has been developed within the framework of the broad class of *M*-estimators, future research could explore the behavior of the MSPE for other estimators and model classes. These could involve further misspecifications, small sample contexts, complex data designs such as hierarchical data and complex time series analyses. Furthermore, estimators using limited information, such as generalized method of moments approaches (Hansen, 1982), could be examined. Many of these methods using limited information are characterized by asymptotic standard errors that provide accurate approximations of parameter estimate variability primarily for larger sample sizes. Additionally, exploring the potential of bootstrapping as a means to mitigate limitations associated with asymptotic standard errors in the MSPE approach could offer valuable insights.

## Conclusion

The MSPE method serves as a valuable instrument for modeling power for the *z*-test in cases where analytical power solutions are absent. Its adaptability becomes evident as it accommodates models even when they contain distributional misspecifications. The MSPE showed good performance even under distributional misspecification for linear SEM and QISEM in terms of bias, RMSE, and type I error rates. Consequently, the MSPE can aid study planning in a variety of contexts of complex study and data designs as long as the parameter estimates are asymptotically normal by economically incorporating all correlative information between power rate and sample size.

## Data Availability

All data is available at https://osf.io/6qh4u/.

## Appendix A: Details on asymptotic normality of *M*-estimators used for power estimation

Here, we provide some more detailed definitions regarding *M*-estimators (see, e.g., Wooldrige, 2010, Chapter 12).

### Asymptotic normality of *M*-estimators

For a *q*-dimensional data vector $\varvec{Y}$ and an objective function $\mathcal {Q}$, that depends on the data and on a *p*-dimensional parameter vector $\vartheta _0$ (population values of the parameters), the *M*-estimator for $\vartheta _0$ is defined for *n* realizations (a sample of size *n*) on a parameter space $\Theta \subset \mathbb {R}^p$ (the parameters $\vartheta$ lie in $\Theta$) as

A1 $$\begin{aligned} \hat{\vartheta }_n = \arg \min _{\vartheta \in \Theta } \frac{1}{n}\sum _{i=1}^n\mathcal {Q}(\varvec{Y}_i,\vartheta ). \end{aligned}$$

$\mathcal {Q}$ can have very different shapes. Examples are the sum of squares of the residuals (OLS estimation), or the negative sum of the log-likelihood values (ML estimation). $\hat{\vartheta }_n$ is assumed to be unique, which can be formalized by assuming that $\vartheta _0$ uniquely solves

$$ \vartheta _0 = \arg \min _{\vartheta \in \Theta } \mathbb {E}\left[ \mathcal {Q}(\varvec{Y},\vartheta )\right] , $$

where $\mathbb {E}$ denotes the expected value. By the law of large numbers the average objective function converges to the expected objective function $\frac{1}{n}\sum _{i=1}^n\mathcal {Q}(\varvec{Y}_i,\vartheta )\overset{\mathbb {P}}{\underset{n\rightarrow \infty }{\longrightarrow }}\mathbb {E}\left[ \mathcal {Q}(\varvec{Y},\vartheta )\right]$ under rather week moment conditions, where $\overset{\mathbb {P}}{\underset{n\rightarrow \infty }{\longrightarrow }}$ denotes convergence in probability. This can be used to prove

$$ \hat{\vartheta }_n\overset{\mathbb {P}}{\underset{n\rightarrow \infty }{\longrightarrow }}\vartheta _0, $$

which states that the *M*-estimator converges towards the true parameter vector under some regularity conditions (see, e.g., Wooldrige, 2010) in probability.

Under some more regularity conditions, including the positive definiteness of the variance of the score function and the hessian of the objective function with regard to the parameters, the *M*-estimator is asymptotically normally distributed

A2 $$\begin{aligned} \sqrt{n} (\hat{\vartheta }_n - \vartheta _0) \overset{d}{\underset{n\rightarrow \infty }{\longrightarrow }}\mathcal {N}\left( 0, \mathcal {H}^{-1}\mathcal {I} \mathcal {H}^{-1}\right) , \end{aligned}$$

where $\overset{d}{\underset{n\rightarrow \infty }{\longrightarrow }}$ denotes convergence in distribution and $\mathcal {N}\left( 0, \mathcal {H}^{-1}\mathcal {I} \mathcal {H}^{-1}\right)$ is the multivariate normal distribution with zero mean and covariance matrix $\mathcal {H}^{-1}\mathcal {I} \mathcal {H}^{-1}$. Here, $\mathcal {I}$ is the variance of the score function (under some further conditions this matrix is called the Fisher information matrix) and $\mathcal {H}$ is the expected Hessian of the objective function evaluated at $\vartheta _0$, respectively,

$$\begin{aligned} \mathcal {I}&= \mathbb {E}\left[ \mathcal {S}(\varvec{Y},\vartheta _0)\mathcal {S}(\varvec{Y},\vartheta _0)'\right] =\mathbb {V}ar \, \left[ \mathcal {S}(\varvec{Y},\vartheta _0)\right] ,\\ \mathcal {H}&= \mathbb {E}\left[ \ddot{\mathcal {H}}(\varvec{Y},\vartheta _0)\right] , \end{aligned}$$

with $\mathcal {S}$ being the score function, consisting of the gradient of the objective function, evaluated at $\vartheta _0$, and $\ddot{\mathcal {H}}(\varvec{Y},\vartheta _0)$ is the Hessian of the objective function $\mathcal {Q}(\varvec{Y},\vartheta _0)$, evaluated at $\vartheta _0$ (Wooldrige, 2010):

$$\begin{aligned} \mathcal {S}(\varvec{Y},\vartheta _0)&=\nabla _{\vartheta _0} \mathcal {Q}(\varvec{Y},\vartheta _0),\\ \ddot{\mathcal {H}}(\varvec{Y},\vartheta _0)&=\nabla ^2_{\vartheta _0} \mathcal {Q}(\varvec{Y},\vartheta _0). \end{aligned}$$

The first derivatives constituting the Jacobian with respect to $\vartheta _0$ are denoted by $\nabla _{\vartheta _0}$. The second order derivatives constituting the Hessian is denoted by $\nabla _{\vartheta _0}^2$. The square root of the diagonal elements of $AVAR(\hat{\vartheta }_n)=\frac{1}{n}\mathcal {H}^{-1}\mathcal {I} \mathcal {H}^{-1}$ correspond to the standard errors of the parameters. Under some regularity conditions the matrices can be replaced by their sample counterparts estimated from the sample of size *n* (hence, $\widehat{AVAR}(\hat{\vartheta }_n)=\frac{1}{n}\hat{\mathcal {H}}^{-1}\hat{\mathcal {I}} \hat{\mathcal {H}}^{-1}$ evaluated at $\hat{\vartheta }_n$). From the asymptotic normality results

$$\hat{\vartheta }_n \overset{a}{\sim }\ \mathcal {N}\left( \vartheta _0, \frac{1}{n} \mathcal {H}^{-1}\mathcal {I} \mathcal {H}^{-1}\right) , $$

hence, $\mathbb {E}\left[ \hat{\vartheta }_n\right] =\vartheta _0$, for large *n*.

We call $\hat{\vartheta }_{n,j}$ the *j*-th entry of $\hat{\vartheta }_n$ and its standard error $SE(\hat{\vartheta }_{n,j}) = \sqrt{AVAR(\hat{\vartheta }_n)_{j,j}}$. For a predefined type I error rate $\alpha$, we define the critical value under normality as the positive quantile $q_\text (1-\alpha )$, so that the probability of a standard normally distributed variable to be larger than $q_\text (1-\alpha )$ is $\alpha$. Then the *z*-value $\mathcal {Z}_n$ is computed as the ratio of the estimate divided by its standard error:

A3 $$\begin{aligned} \mathcal {Z}_n&= \frac{\hat{\vartheta }_{n,j}}{SE(\hat{\vartheta }_{n,j})},\quad \text { and }\nonumber \\ \mathbb {E}\left[ \mathcal {Z}_n \right]&= \frac{\vartheta _{0,j}}{SE(\hat{\vartheta }_{n,j})} = \sqrt{n}\cdot \frac{\vartheta _{0,j}}{\sqrt{\left( \mathcal {H}^{-1}\mathcal {I}\mathcal {H}^{-1}\right) _{j,j}}} \end{aligned}$$

Hence, under the null hypothesis $H_0:\vartheta _{0,j}=0$, $\mathbb {E}[\mathcal {Z}^{H_0}_n] = 0$, and the event that $\left\{ \mathcal {Z}^{H_0}_n>q_\text (1-\alpha )\right\}$ should have a probability of $\alpha$ for large *n*. Under $H_1$, $\vartheta _{0,j}\ne 0$ and, consequently,

$$ \mathcal {Z}^{H_1}_n \overset{a}{\sim }\mathcal {N}\left( \sqrt{n}\cdot \frac{\vartheta _{0,j}}{\sqrt{\left( \mathcal {H}^{-1}\mathcal {I}\mathcal {H}^{-1}\right) _{j,j}}},1\right) , $$

which has a diverging mean (i.e., $\sqrt{n}\cdot \frac{|\vartheta _{0,j}|}{\sqrt{\left( \mathcal {H}^{-1}\mathcal {I}\mathcal {H}^{-1}\right) _{j,j}}}\underset{n\rightarrow \infty }{\longrightarrow }\infty$).

Now, in order to estimate power, we need to study the event of a significant effect. Since $\vartheta _{0,j}$ may have a positive or negative sign, this needs to be taken into account when computing a one-sided test by using the sign of $\vartheta _{0,j}$

$$\left\{ sign(\vartheta _{0,j})\mathcal {Z}^{H_1}_n>q_\text (1-\alpha )\right\} . $$

For $\vartheta _{0,j}>0,\ sign(\vartheta _{0,j})=1$ and there is nothing to show; for $\vartheta _{0,j}<0,\ sign(\vartheta _{0,j})=-1$ we study the event

$$\begin{aligned} \left\{ -\mathcal {Z}^{H_1}_n>q_\text (1-\alpha )\right\} = \left\{ \mathcal {Z}^{H_1}_n< -q_\text (1-\alpha )\right\} = \left\{ \mathcal {Z}^{H_1}_n<q_\text (\alpha )\right\} , \end{aligned}$$

by using that $q_\text (1-\alpha ) = -q_\text (\alpha )$. Now, $\left\{ \mathcal {Z}^{H_1}_n<q_\text (\alpha )\right\}$ is the event of interest coding a significant effect if the true effect is expected to be negative.

## Appendix B: Details and derivations for the MSPE for one-sided and two-sided *z*-tests

From Appendix A, it follows that the probability of the event that $\left\{ sign(\vartheta _{0,j})\mathcal {Z}^{H_1}_n>q_\text (1-\alpha )\right\}$ should have a probability (much) larger than $\alpha$ for large *n* under $H_1$. As we know the asymptotic distribution and its mean of the estimate divided by its standard error, we make use of this in order to describe the probability of the event $\left\{ sign(\vartheta _{0,j})\mathcal {Z}^{H_1}_n>q_\text (1-\alpha )\right\}$ by approximating it with the value of the distribution function $\Phi$ of the standard normal distribution by defining a standard normal variable $Z\sim \mathcal {N}(0,1)$ for which we have that asymptotically $\mathcal {Z}^{H_1}_n=\frac{\hat{\vartheta }_{n,j}}{SE(\hat{\vartheta }_{n,j})}\overset{d}{=}\ Z + \frac{\vartheta _{0,j}}{SE(\hat{\vartheta }_{n,j})}$, where equality in distribution is denoted by $\overset{d}{=}$. Therefore, we can derive the asymptotic probability

$$\begin{aligned} \mathbb {P}&\left( sign(\vartheta _{0,j})\mathcal {Z}^{H_1}_n> q (1-\alpha )\right) \\&\approx \mathbb {P}\left( sign(\vartheta _{0,j})Z+sign(\vartheta _{0,j})\frac{\vartheta _{0,j}}{SE(\hat{\vartheta }_{n,j})}>q_\text (1-\alpha )\right) \\&= \mathbb {P}\left( sign(\vartheta _{0,j})Z>q (1-\alpha )-\frac{sign(\vartheta _{0,j})\vartheta _{0,j}}{\sqrt{\frac{1}{n}\left( \mathcal {H}^{-1}\mathcal {I}\mathcal {H}^{-1}\right) _{j,j}}}\right) \\&= \mathbb {P}\left( Z>q(1-\alpha )-\frac{|\vartheta _{0,j}|}{\sqrt{\left( \mathcal {H}^{-1}\mathcal {I}\mathcal {H}^{-1}\right) _{j,j}}}\sqrt{n}\right) , \end{aligned}$$

since $Z\overset{d}{=}-Z$, as the standard normal distribution is symmetric and, therefore, $sign(\vartheta _{0,j})Z \overset{d}{=}Z$, and $sign(\vartheta _{0,j})\vartheta _{0,j} = |\vartheta _{0,j}|$ by the definition of the absolute value. Now we use that $\mathbb {P}(Z>a)$ $=1-\mathbb {P}(Z\le a)$ $= 1-\Phi (a)$, for standard normally distributed *Z*, where $\Phi$ is the cumulative density function of the standard normal distribution. Therefore,

$$\begin{aligned} \mathbb {P}&\left( Z>q (1-\alpha )-\frac{|\vartheta _{0,j}|}{\sqrt{\left( \mathcal {H}^{-1}\mathcal {I}\mathcal {H}^{-1}\right) _{j,j}}}\sqrt{n}\right) \\&=1-\Phi \left( q (1-\alpha )- \frac{|\vartheta _{0,j}|}{\sqrt{\left( \mathcal {H}^{-1}\mathcal {I}\mathcal {H}^{-1}\right) _{j,j}}}\sqrt{n}\right) \\&=\Phi \left( -q (1-\alpha )+\frac{|\vartheta _{0,j}|}{\sqrt{\left( \mathcal {H}^{-1}\mathcal {I}\mathcal {H}^{-1}\right) _{j,j}}}\sqrt{n}\right) . \end{aligned}$$

The last line follows from the symmetry of the standard normal distribution: $\Phi (a)=1-\Phi (-a)$.

### Fitting a probit regression to the significance decisions

Let $\mathcal {S}_i = \textbf{1}_{\left\{ sign(\vartheta _{0,j})\mathcal {Z}^{H_1}_{n_i}>q_\text (1-\alpha )\right\} }$ be the indicator function which codes the significance decision for $i=1,\dots ,R$ simulations, where

$$ \mathcal {Z}^{H_1}_{n_i} = \frac{\hat{\vartheta }_{n_i}}{SE(\hat{\vartheta }_{n_i})}. $$

Hence, $\mathcal {S}_i = 1$ if the *i*-th replication yielded a significant result and $\mathcal {S}_i = 0$ else. Then the probability of a significant result given a sample of size $n_i$ is given by, for $i=1,\dots ,R$,

$$\begin{aligned}&\mathbb {P}\left( \mathcal {S}_i = 1 | n_i, \beta _0, \beta _1 \right) = \Phi (\beta _0 + \beta _1 \sqrt{n_i}), \\&\quad \beta _\text {probit}=(\beta _0,\beta _1)'=\left( -q_\text (1-\alpha ),\frac{\left| \vartheta _{0,j}\right| }{\sqrt{\left( \mathcal {H}^{-1}\mathcal {I}\mathcal {H}^{-1}\right) _{j,j}}}\right) '. \end{aligned}$$

Hence, it is obvious that approximately, the significance decision follows a probit model with the square root of the sample size ($\sqrt{n}$) as a predictor. As $\sqrt{\left( \mathcal {H}^{-1}\mathcal {I}\mathcal {H}^{-1}\right) _{j,j}}$ is a constant, it would be possible to retrieve it asymptotically from the estimated $\beta$-coefficient in this probit model.

### Minimal *n*

In order to solve for the minimal *n* required to result in the desired power $\rho$ for a given $\alpha$ level, we invert the probit regression link function and solve for *n*, if $\mathcal {I}$ and $\mathcal {H}$ cannot be derived analytically.

$$\begin{aligned} \rho&= \Phi \left( -q(1-\alpha )+\frac{|\vartheta _{0,j}|}{\sqrt{\left( \mathcal {H}^{-1}\mathcal {I}\mathcal {H}^{-1}\right) _{j,j}}}\sqrt{n}\right) \\&\Longleftrightarrow \frac{\Phi ^{-1}(\rho ) + q (1-\alpha )}{\frac{|\vartheta _{0,j}|}{\sqrt{\left( \mathcal {H}^{-1}\mathcal {I}\mathcal {H}^{-1}\right) _{j,j}}}} = \sqrt{n}\\&\Longleftrightarrow \left( \frac{\Phi ^{-1}(\rho ) + q (1-\alpha )}{\frac{|\vartheta _{0,j}|}{\sqrt{\left( \mathcal {H}^{-1}\mathcal {I}\mathcal {H}^{-1}\right) _{j,j}}}}\right) ^{2} = n \end{aligned}$$

Now, *n* needs to be an integer that is larger or equal to the value derived in the last line. Hence, we round up to the next integer and call this required sample size $N_\alpha$

$$\begin{aligned} N_\alpha&\approx \left\lceil \left( \frac{\Phi ^{-1}(\rho ) + q_\text (1-\alpha )}{\frac{|\vartheta _{0,j}|}{\sqrt{\left( \mathcal {H}^{-1}\mathcal {I}\mathcal {H}^{-1}\right) _{j,j}}}}\right) ^2\right\rceil \\&\quad =\left\lceil \left( \Bigg (\Phi ^{-1}(\rho ) + q_\text (1-\alpha ) \Bigg ) \cdot \left( \frac{|\vartheta _{0,j}|}{\sqrt{\left( \mathcal {H}^{-1}\mathcal {I}\mathcal {H}^{-1}\right) _{j,j}}}\right) ^{-1}\right) ^2\right\rceil . \end{aligned}$$

$N_\alpha$ can also be expressed in terms of the probit regression coefficients:

$$ N_\alpha \approx \left\lceil \left( \frac{\Phi ^{-1}(\rho ) -\beta _0}{\beta _1}\right) ^2\right\rceil . $$

Since $N_\alpha$ depends on the random process underlying the significance decision, it is a random variable with a distribution and, therefore, subject to variability. In order to ensure that the computed sample size yields the desired power $\rho$, we utilize the $1-\alpha _\rho$ normal-theory confidence band around $\rho$, namely, $[\hat{\rho }_\text {lb},\hat{\rho }_\text {ub}]$, where $\hat{\rho }_\text {lb}$ and $\hat{\rho }_\text {ub}$ are the lower and upper bound of the confidence interval around the required sample size. If we choose the lower bound to equal $\rho$, and select the corresponding predicted power rate $\hat{\rho }_{\hat{\rho }_\text {ub}=\rho }$, this ensures that

$$\begin{aligned} N_{\alpha ,\text {lb}}&\approx \left\lceil \left( \frac{\Phi ^{-1}(\hat{\rho }_{\hat{\rho }_\text {ub}=\rho }) -\beta _0}{\beta _1}\right) ^2\right\rceil \\&\quad \!=\! \left\lceil \left( \Bigg (\Phi ^{-1}(\hat{\rho }_{\hat{\rho }_\text {ub}=\rho }) \!+\! q_\text (1\!-\!\alpha ) \Bigg ) \cdot \left( \frac{|\vartheta _{0,j}|}{\sqrt{\left( \mathcal {H}^{-1}\mathcal {I}\mathcal {H}^{-1}\right) _{j,j}}}\right) ^{-1}\right) ^2\right\rceil , \end{aligned}$$

to be larger or equal to $N_\alpha$ with probability $1-\alpha _\rho /2$.

### Two-sided test and Wald test

Instead of a one-sided test per parameter, a two-sided test can be computed, which in this case is equivalent to the Wald test. Since it is a two-sided test, the event of interest is $\left\{ \left| \mathcal {Z}^{H_1}_n\right| >q_\text (1-\alpha /2)\right\}$, with probability

$$\begin{aligned} \mathbb {P}&\left( \left| \mathcal {Z}^{H_1}_n\right|>q (1-\alpha /2)\right) \\&= \mathbb {P}\left( \mathcal {Z}^{H_1}_n>q (1-\alpha /2)\right) +\mathbb {P}\left( \mathcal {Z}^{H_1}_n<-q (1-\alpha /2)\right) \nonumber \\&\approx \mathbb {P}\left( Z+\frac{\vartheta _{0,j}}{SE(\hat{\vartheta }_{n,j})}>q (1-\alpha /2)\right) \\&\qquad + \mathbb {P}\left( Z+ \frac{\vartheta _{0,j}}{SE(\hat{\vartheta }_{n,j})}< - q (1-\alpha /2)\right) \nonumber \\&= \mathbb {P}\left( Z>q (1-\alpha /2) - \frac{\vartheta _{0,j}}{SE(\hat{\vartheta }_{n,j})}\right) \\&\qquad + \mathbb {P}\left( Z<-q (1-\alpha /2)- \frac{\vartheta _{0,j}}{SE(\hat{\vartheta }_{n,j})}\right) . \end{aligned}$$

Hence, for $Z\sim \mathcal {N}(0,1)$, we have

B1 $$\begin{aligned} \mathbb {P}&\left( \left| \mathcal {Z}^{H_1}_n\right| >q_\text (1-\alpha /2)\right) \\&= \Phi \left( -q_\text (1-\alpha /2)+\frac{\vartheta _{0,j}}{\sqrt{\left( \mathcal {H}^{-1}\mathcal {I}\mathcal {H}^{-1}\right) _{j,j}}}\sqrt{n}\right) \nonumber \\&\quad +\Phi \left( -q_\text (1-\alpha )-\frac{\vartheta _{0,j}}{\sqrt{\left( \mathcal {H}^{-1}\mathcal {I}\mathcal {H}^{-1}\right) _{j,j}}}\sqrt{n}\right) ,\nonumber \end{aligned}$$

where for large *n* one of the two expression converges to zero, while the other converges to 1, depending on the sign of $\vartheta _{0,j}$. $N_\alpha$ can be computed by numerically solving for *n*.

#### Estimation of two-sided probit model

For the probability in Eq. B1 of the two-sided test, a variant of a probit model can be stated, since for $\beta ^\circ _0 = -q_\text (1-\alpha /2)$ and $\beta _1^\circ = \frac{\vartheta _{0,j}}{\sqrt{\left( \mathcal {H}^{-1}\mathcal {I}\mathcal {H}^{-1}\right) _{j,j}}}$, for $\beta ^\circ = (\beta ^\circ _0, \beta ^\circ _1)'$ Eq. B1 is, where we write $\mathcal {S}_i^\circ = \textbf{1}_{\left\{ \left| \mathcal {Z}^{H_1}_n\right| >q_\text (1-\alpha /2)\right\} }$ for the indicator function coding a significant result,

B2 $$\begin{aligned} \mathbb {P}(\mathcal {S}_i^\circ = 1 | n_i, \beta ^\circ ) = \Phi (\beta ^\circ _0 + \beta ^\circ _1\sqrt{n_i}) + \Phi (\beta ^\circ _0 - \beta ^\circ _1\sqrt{n_i}). \end{aligned}$$

The likelihood is then stated as

B3 $$\begin{aligned}&\mathcal {L}(\mathcal {S}_1^\circ ,\dots ,\mathcal {S}_R^\circ , n_1, \dots , n_R,\beta ^\circ )\nonumber \\&\quad = \prod _{i=1}^R\mathbb {P}(\mathcal {S}_i^\circ = 1 | n_i, \beta ^\circ )^{\mathcal {S}_i^\circ }(1-\mathbb {P}(\mathcal {S}_i^\circ = 1 | n_i, \beta ^\circ ))^{1-\mathcal {S}_i^\circ }. \end{aligned}$$

$\beta ^\circ$ is then estimated by maximizing the log-likelihood of the significance decision across the *R* replications:

$$\hat{\beta }^\circ = \arg \max _{\beta ^\circ \in \mathbb {R}^2} \log \left( \mathcal {L}(\mathcal {S}_1^\circ ,\dots ,\mathcal {S}_R^\circ , n_1, \dots , n_R,\beta ^\circ )\right) , $$

where $\mathbb {R}$ is the set of real numbers. A normal-theory-based confidence interval can be computed for $\mathbb {P}\left( \mathcal {S}^\circ = 1 | n, \hat{\beta }^\circ \right)$ by using that the estimates $\hat{\beta }^\circ$ are asymptotically normal with variance $AVAR(\hat{\beta }^\circ )$. Let $\mathbb {P}_\text {lb}\left( \mathcal {S}^\circ = 1 | n, \hat{\beta }^\circ \right)$ be the lower bound of the $1-\alpha _\rho$ interval of confidence for $\mathbb {P}\left( \mathcal {S}^\circ = 1 | n, \hat{\beta }^\circ \right)$, then $N_{\alpha ,\text {lb}}$ is derived numerically via

$$ N_{\alpha ,\text {lb}} = \min _{n\in \mathbb {N}} \left\{ \mathbb {P}_\text {lb}\left( \mathcal {S}^\circ = 1 | n, \hat{\beta }^\circ \right) \ge \rho \right\} . $$

## Appendix C: Additional information on simulation studies

### Simulation conditions for linear SEM

The model is given in Fig. 2. The structural model is

$$\begin{aligned} \eta _1&= .2\xi _1 + .5\xi _2 + \zeta _1,\\ \eta _2&= .3\eta _1 + .4\xi _1 + .3\xi _2 + \zeta _2, \end{aligned}$$

where the variances of $\zeta _1$ and $\zeta _2$ were set so that all latent variables ($\xi _1,\xi _2,\eta _1,\eta _2$) were standardized and $\xi _1$ and $\xi _2$ were correlated by .5. All latent variables were measured by three indicator variables and the overall reliability (estimated using McDonald’s $\omega$, McDonald, 1999) was set to be rather high ($\Lambda =[1,.8,.7]', \omega =.85$). The following elements are rounded to max 4 decimals.

$$\begin{aligned} \Lambda _x =\Lambda _y&= \begin{bmatrix} 1 & 0 \\ .8 & 0 \\ .7 & 0 \\ 0 & 1 \\ 0 & .8 \\ 0 & .7 \end{bmatrix},\\ \mathbb {C}ov \, \varvec{\xi } = \Phi = \begin{bmatrix} 1 & \\ \phi _{21} & 1 \end{bmatrix}&= \begin{bmatrix} 1 & \\ .5 & 1 \end{bmatrix},\\ \mathbb {V}ar \, \varvec{\zeta } =\Psi = \begin{bmatrix} \psi _{11} & \\ 0 & \psi _{22} \end{bmatrix}&= \begin{bmatrix} .61 & \\ 0 & .324 \end{bmatrix}, \end{aligned}$$

where $\varvec{\zeta }$ and $\varvec{\xi }$ are independent. The measurement models are given by

$$\begin{aligned} \varvec{X}&= \Lambda _x \varvec{\xi } + \varvec{\delta },\\ \varvec{Y}&= \Lambda _y \varvec{\eta } + \varvec{\varepsilon }, \end{aligned}$$

with $\varvec{\xi }=(\xi _1,\xi _2)', \varvec{\eta }=(\eta _1,\eta _2)'$. The covariance matrices of the residuals are given by

$$\begin{aligned} \Theta _x = \mathbb {C}ov \, \varvec{\delta }&= \text {diag}(.2345, .36, .51,.2345, .36, .51),\\ \Theta _y = \mathbb {C}ov \, \varvec{\varepsilon }&= \text {diag}(.2345, .36, .51,.2345, .36, .51), \end{aligned}$$

where $\text {diag}(v)$ creates a diagonal matrix with diagonal elements *v*. The measurement errors $\varvec{\delta }$ and $\varvec{\varepsilon }$ are independent to all other variables in the model. The corresponding item-level reliabilites were .81, .64, .49, .81, .64,  and .49 for the elements of $\varvec{X}$, respectively, and .81, .64, .49, .81, .64,  and .49 for $\varvec{Y}$, respectively.

The corresponding formulas for Fig. 2 are

$$\begin{aligned} \eta _1&= \gamma _{11}\xi _1 + \gamma _{12}\xi _2 + \zeta _1,\\ \eta _2&= \beta _{21}\eta _1 + \gamma _{21}\xi _1 + \gamma _{22}\xi _2 + \zeta _2, \end{aligned}$$

in LISREL notation (Jöreskog, 1970).

The model-implied covariance matrix is given by

$$ \Sigma = \begin{bmatrix} \Lambda _x \Phi \Lambda _x' + \Theta _x & & & & & \Lambda _x \Phi \Gamma ' ((\text {I}-\text {B})^{-1})'\Lambda _y' \\ \Lambda _y (\text {I}-\text {B})^{-1}\Gamma \Phi \Lambda _x' & & & & & \Lambda _y (\text {I}-\text {B})^{-1}\left( \Gamma \Phi \Gamma '+\Psi \right) ((\text {I}-\text {B})^{-1})' \Lambda _y' + \Theta _y \end{bmatrix},$$

where $\text {I}$ is the identity matrix, $\Gamma$ is the matrix collecting the effects between the exogenous variables, and $\text {B}$ is the matrix collecting the effects between the exogenous and the endogenous side of the model, and see, e.g., Jöreskog (1970). All means were set to 0, hence, in the normality condition the data were distributed as follows

$$ \begin{pmatrix} \varvec{X} \\ \varvec{Y} \end{pmatrix} \sim \mathcal {N}(\varvec{0}, \Sigma ). $$

For the non-normal condition the Vale and Maurelli (1983) approach was used with the following (univariate) skewness $\mathbb {S}\text {kew}$ and (univariate excess) kurtosis $\mathbb {K}\text {urt}$

$$\begin{aligned} \mathcal {S}_x&= \mathbb {S}\text {kew}[\varvec{X}] = (.7, 1, 1.3,.7, 1, 1.3)', \quad &  \mathcal {S}_y = \mathbb {S}\text {kew}[\varvec{Y}] = (1.7, 2, 2.3,1.7, 2, 2.3)', \\ \mathcal {K}_x&= \mathbb {K}\text {urt}[\varvec{X}] = (3.2, 2.9, 2.5,3.2, 2.9, 2.5)', &  \mathcal {K}_y = \mathbb {K}\text {urt}[\varvec{Y}] = (7.2, 6.9, 6.5,7.2, 6.9, 6.5)', \end{aligned}$$

which utilizes third-degree polynomials, so-called Fleishman (1978) polynomials, of correlated normal variables to generate non-normal data with predefined univariate skewness and kurtosis. Hence, the corresponding Vale and Maurelli (1983) distribution is

$$ \begin{pmatrix} \varvec{X} \\ \varvec{Y} \end{pmatrix} \sim \mathcal{V}\mathcal{M}\left( \varvec{0}, \Sigma , \begin{pmatrix} \mathcal {S}_x \\ \mathcal {S}_y \end{pmatrix}, \begin{pmatrix} \mathcal {K}_x \\ \mathcal {K}_y \end{pmatrix}\right) , $$

where $\mathcal{V}\mathcal{M}\left( m,S,s, k\right)$ is the Vale and Maurelli (1983) distribution with mean *m*, covariance matrix *S*, and univariate skewness *s* and kurtosis *k*.

### Simulation conditions for QISEM

For the QISEM, the linear SEM of the previous section is extended to

$$\begin{aligned} \eta _1&= .2\xi _1 + .5\xi _2 + .1\xi _1\xi _2 + \zeta _1,\\ \eta _2&= .3\eta _1 + .4\xi _1 + .3\xi _2 + \zeta _2, \end{aligned}$$

and the residual variances where set to

$$\begin{aligned} \mathbb {V}ar \, \varvec{\zeta } =\Psi = \begin{bmatrix} \psi _{11} & \\ 0 & \psi _{22} \end{bmatrix}&= \begin{bmatrix} .5975 & \\ 0 & .3251 \end{bmatrix}, \end{aligned}$$

in order for all latent variables ($\xi _1,\xi _2,\eta _1,\eta _2$) to be standardized. All other parameters were set identically to the linear SEM of the previous section. In the normal condition, $\xi _1,\xi _2, \zeta _1,\zeta _2,\varvec{\delta },\varvec{\varepsilon }$ were simulated to be normal. The latent dependent variables and their indicators differ from normality only through the model-implied non-normality of the interaction effect. For the non-normal condition, $\zeta _1,\zeta _2,\varvec{\delta },\varvec{\varepsilon }$ were simulated to be normal, and $\xi _1,\xi _2$ were set to be Vale and Maurelli (1983) distributed as

$$ \begin{pmatrix}\xi _1 \\ \xi _2\end{pmatrix} \sim \mathcal{V}\mathcal{M}\left( \begin{pmatrix} 0\\ 0 \end{pmatrix}, \begin{bmatrix} 1 & .5 \\ .5 & 1 \end{bmatrix}, \begin{pmatrix} 1.7\\ 2.3 \end{pmatrix}, \begin{pmatrix} 7.2 \\ 6.5 \end{pmatrix} \right) . $$

## Appendix D: Additional graphics

Figure 8 illustrates the power rates predicted by the MSPE and by linear interpolation in the model setting of Simulation Study 2 (for more details the Figure note). Additionally, the required sample sizes are computed and compared to the reference sample size of 700, which corresponds to a power rate of $\rho \approx .812$, which is the simulated power rate for $n=700$ based on $10^6$ simulated data sets. The results indicate that the MSPE, even when based on just four different sample sizes, is a more efficient method for modeling power compared to the naïve approach of selecting distinct sample sizes as it uses the correct parametric form and all simulated data sets. The sample sizes determined by the MSPE were $\hat{N}_{\alpha , \text {MSPE}} = 689$ and $\hat{N}_{\text {lb}, \alpha , \text {MSPE}} = 725$, while those determined by linear interpolation were $\hat{N}_{\alpha , }$_naïve_ $= 721$ and $\hat{N}_{\text {lb}, \alpha , }$_naïve_ $= 811$. The MSPE provided closer approximations to the reference sample size of 700.

The discrepancy between the *z*-test and the LRT is illustrated in Fig. 9 by non-overlapping confidence bands for the linear SEM model used in the first simulation study. The analytical asymptotic power rates depicted in Fig. 9 were computed using the semPower package (Moshagen & Bader, 2024), which utilizes the asymptotic power estimates proposed by Satorra and Sarris (1985) and MacCallum et al. (1996). These approaches approximate power for the LRT by estimating a non-centrality parameter, quantified by the expected difference in the LRT (Satorra & Saris, 1985; Satorra, Saris, & de Pijper, 1991), or the root mean square error of approximation (RMSEA, MacCallum et al., 1996).

## Appendix E: Additional tables

**Table 2:** Type I error rates for SEM estimation for selected sample sizes

Dist	n	$Z_\text {one}$	lb: $Z_\text {one}$	ub: $Z_\text {one}$	$Z_\text {two}$	lb: $Z_\text {two}$	ub: $Z_\text {two}$	LRT	lb: LRT	ub: LRT	Conv
norm	50	0.063	0.062	0.063	0.064	0.064	0.065	0.061	0.061	0.062	0.833
norm	350	0.052	0.052	0.053	0.052	0.052	0.052	0.051	0.051	0.052	1.000
norm	650	0.051	0.051	0.052	0.051	0.050	0.051	0.050	0.050	0.051	1.000
VM	50	0.058	0.057	0.058	0.066	0.065	0.066	0.025	0.025	0.026	0.720
VM	350	0.048	0.048	0.049	0.053	0.052	0.053	0.050	0.050	0.051	1.000
VM	650	0.048	0.047	0.048	0.052	0.051	0.052	0.051	0.051	0.051	1.000

*Note.* Dist = distributional condition (norm = normally distributed, VM = Vale-Maurelli distribution), $Z_\text {one}$ = one-sided *z*-test, $Z_\text {two}$ = two-sided *z*-test, LRT = (robust) likelihood ratio test, lb = lower bound of confidence interval, ub = upper bound of confidence interval, Conv = convergence rate

**Table 3:** Type I error rates for QISEM estimation for selected sample sizes

Dist	n	$Z_\text {one}$	lb: $Z_\text {one}$	ub: $Z_\text {one}$	$Z_\text {two}$	lb: $Z_\text {two}$	ub: $Z_\text {two}$	LRT	lb: LRT	ub: LRT	Conv
norm	150	0.054	0.054	0.055	0.057	0.057	0.058	0.020	0.020	0.021	0.891
norm	675	0.051	0.051	0.052	0.052	0.051	0.052	0.041	0.040	0.041	0.998
norm	1200	0.050	0.050	0.051	0.051	0.050	0.051	0.045	0.044	0.045	1.000
VM	150	0.072	0.071	0.072	0.081	0.081	0.082	0.024	0.024	0.024	0.946
VM	675	0.065	0.065	0.066	0.072	0.072	0.073	0.038	0.038	0.039	0.999
VM	1200	0.062	0.061	0.062	0.068	0.067	0.068	0.042	0.041	0.042	1.000

*Note.* Dist = distributional condition (norm = normally distributed, VM = Vale-Maurelli distribution), $Z_\text {one}$ = one-sided *z*-test, $Z_\text {two}$ = two-sided *z*-test, LRT = (robust) likelihood-ratio test, lb = lower bound of confidence interval, ub = upper bound of confidence interval, Conv = convergence rate

**Table 4:** Performance of the MSPE and alternative methods for linear SEM for $R=2000$ for one-sided *z*-test for Selected reference sample sizes

Dist	Model	$\hat{\rho }_{\text {ref},N_\alpha }$	$\hat{\rho }_{N_\alpha }$	$N_\alpha$	$M(\hat{N}_{\alpha , \star })$	$SD(\hat{N}_{\alpha , \star })$	$min(\hat{N}_{\alpha , \star })$	$max(\hat{N}_{\alpha , \star })$	$M(\hat{N}_{\alpha , \text {lb}})$	Type I
norm	Logit	0.349	0.409	50	26.23	11.83	1	56	47.31	0.559
norm	Logit	0.801	0.790	210	216.87	6.64	195	235	230.21	0.001
norm	Logit	0.857	0.851	250	255.22	7.50	231	278	270.85	0.003
norm	Logit	0.898	0.897	290	291.73	8.83	266	320	310.51	0.012
norm	Logit	0.989	0.991	530	512.03	20.59	457	576	556.80	0.116
norm	Logit-sqrt	0.349	0.312	50	58.62	6.10	40	76	70.09	0.000
norm	Logit-sqrt	0.801	0.816	210	201.08	6.39	181	219	214.09	0.244
norm	Logit-sqrt	0.857	0.869	250	240.14	7.49	216	262	256.23	0.193
norm	Logit-sqrt	0.898	0.906	290	280.50	9.13	253	310	301.03	0.118
norm	Logit-sqrt	0.989	0.984	530	590.95	29.37	518	691	661.29	0.000
norm	Naïve	0.349	0.349	50	53.36	4.63	50	73	65.93	0.000
norm	Naïve	0.801	0.775	210	220.24	5.57	206	252	234.99	0.000
norm	Naïve	0.857	0.849	250	261.70	17.12	225	307	296.78	0.002
norm	Naïve	0.898	0.877	290	319.01	12.69	266	353	348.55	0.000
norm	Naïve	0.989	0.984	530	545.34	20.97	416	599	579.63	0.000
norm	Probit	0.349	0.447	50	8.76	9.09	1	44	29.51	0.964
norm	Probit	0.801	0.775	210	226.27	6.90	204	245	239.87	0.000
norm	Probit	0.857	0.836	250	266.96	7.58	243	289	282.13	0.000
norm	Probit	0.898	0.885	290	303.68	8.80	277	330	321.39	0.000
norm	Probit	0.989	0.994	530	487.68	18.32	434	548	524.85	0.598
norm	Probit-sqrt	0.349	0.343	50	51.82	6.52	32	70	64.03	0.009
norm	Probit-sqrt	0.801	0.802	210	209.33	6.54	188	228	222.31	0.029
norm	Probit-sqrt	0.857	0.858	250	249.74	7.38	226	271	265.12	0.018
norm	Probit-sqrt	0.898	0.899	290	289.29	8.79	263	317	308.14	0.018
norm	Probit-sqrt	0.989	0.989	530	531.47	23.20	469	606	583.83	0.014
norm	Wald	0.349	0.346	50	51.30	6.96	29	70	63.82	0.012
norm	Wald	0.801	0.802	210	209.44	6.52	188	228	222.45	0.028
norm	Wald	0.857	0.858	250	249.83	7.38	226	271	265.20	0.017
norm	Wald	0.898	0.899	290	289.34	8.79	263	317	308.18	0.018
norm	Wald	0.989	0.989	530	531.13	22.97	469	605	582.69	0.015
VM	Logit	0.306	0.379	50	15.43	11.36	1	63	38.48	0.836
VM	Logit	0.738	0.721	210	220.20	7.04	198	245	233.77	0.000
VM	Logit	0.800	0.787	250	259.12	7.43	236	286	273.91	0.001
VM	Logit	0.848	0.841	290	296.09	8.35	269	326	313.23	0.002
VM	Logit	0.975	0.979	530	510.30	18.20	452	576	549.99	0.162
VM	Logit	0.988	0.991	630	598.91	22.96	525	687	649.35	0.224
VM	Logit-sqrt	0.306	0.282	50	56.68	6.53	33	83	69.20	0.001
VM	Logit-sqrt	0.738	0.750	210	203.92	6.73	183	230	216.86	0.138
VM	Logit-sqrt	0.800	0.811	250	242.02	7.45	220	268	257.06	0.160
VM	Logit-sqrt	0.848	0.857	290	281.31	8.67	254	312	299.70	0.143
VM	Logit-sqrt	0.975	0.969	530	567.08	25.01	494	660	626.66	0.000
VM	Logit-sqrt	0.988	0.982	630	714.44	35.45	608	856	799.70	0.000
VM	Naïve	0.306	0.307	50	53.33	4.65	50	75	67.03	0.000
VM	Naïve	0.738	0.715	210	219.60	6.59	200	248	236.78	0.000
VM	Naïve	0.800	0.789	250	261.05	15.93	223	303	294.18	0.008
VM	Naïve	0.848	0.825	290	314.53	12.58	267	348	343.76	0.000
VM	Naïve	0.975	0.968	530	545.99	18.28	482	599	583.42	0.000
VM	Probit	0.306	0.410	50	3.98	6.10	1	50	21.51	0.989
VM	Probit	0.738	0.710	210	227.35	7.40	204	254	241.40	0.000
VM	Probit	0.800	0.773	250	269.12	7.50	246	296	283.75	0.000
VM	Probit	0.848	0.828	290	307.28	8.21	281	338	323.64	0.000
VM	Probit	0.975	0.983	530	496.72	16.25	441	552	530.50	0.483
VM	Probit	0.988	0.995	630	561.63	19.64	494	633	602.65	0.894
VM	Probit-sqrt	0.306	0.306	50	50.55	6.80	27	79	63.58	0.016
VM	Probit-sqrt	0.738	0.738	210	210.30	6.89	189	236	223.42	0.016
VM	Probit-sqrt	0.800	0.800	250	250.38	7.39	228	277	265.01	0.020
VM	Probit-sqrt	0.848	0.848	290	290.04	8.39	263	321	307.33	0.022
VM	Probit-sqrt	0.975	0.974	530	530.34	20.71	465	606	577.30	0.024
VM	Probit-sqrt	0.988	0.988	630	629.28	27.19	544	736	691.48	0.023
VM	Wald	0.306	0.309	50	49.71	7.40	21	79	63.27	0.024
VM	Wald	0.738	0.738	210	210.52	6.84	190	236	223.56	0.016
VM	Wald	0.800	0.800	250	250.54	7.36	228	277	265.18	0.019
VM	Wald	0.848	0.848	290	290.13	8.41	263	321	307.44	0.022
VM	Wald	0.975	0.975	530	529.90	20.49	465	602	576.13	0.025
VM	Wald	0.988	0.988	630	628.58	26.81	544	729	689.61	0.023

*Note.* Dist = distributional condition (norm = normal distribution, VM = Vale-Maurelli), Model = power model, $\hat{\rho }_{\text {ref}, N_\alpha }$ = computed reference power, $\hat{\rho }_{N_\alpha }$, $N_\alpha$ = reference required sample size, $M(\cdot )$ = mean, $SD(\cdot )$ = standard deviation, $min(\cdot )$ = minimum, $max(\cdot )$ = maximum, $\hat{N}_{\alpha , \star }$ = computed required sample size, $\hat{N}_{\alpha , \text {lb}}$ = computed required sample size using lower bound of power, type I = type I error rate of lower $\hat{N}_{\alpha , \text {lb}}$ being smaller than $N_\alpha$, $N_\alpha$ are selected for $\hat{\rho }_{\text {ref}, N_\alpha }$ to be close to .8 and .9 for the normal condition and to include the smallest and largest selected sample sizes

**Table 5:** Performance of the MSPE and alternative methods for linear SEM for $R=2000$ for two-sided *z*-test for selected reference sample sizes

Dist	Model	$\hat{\rho }_{\text {ref},N_\alpha }$	$\hat{\rho }_{N_\alpha }$	$N_\alpha$	$M(\hat{N}_{\alpha , \star })$	$SD(\hat{N}_{\alpha , \star })$	$min(\hat{N}_{\alpha , \star })$	$max(\hat{N}_{\alpha , \star })$	$M(\hat{N}_{\alpha , \text {lb}})$	Type I
norm	Logit	0.248	0.314	50	17.56	11.22	1	55	38.96	0.828
norm	Logit	0.807	0.797	270	276.80	6.90	258	298	291.13	0.000
norm	Logit	0.877	0.876	330	331.83	8.67	307	362	350.20	0.015
norm	Logit	0.895	0.895	350	349.70	9.40	322	383	369.62	0.025
norm	Logit	0.976	0.980	530	511.68	17.19	462	576	548.16	0.170
norm	Logit-sqrt	0.248	0.222	50	57.90	6.36	37	79	69.52	0.000
norm	Logit-sqrt	0.807	0.819	270	261.25	6.99	242	284	276.19	0.183
norm	Logit-sqrt	0.877	0.883	330	323.21	9.28	298	353	344.00	0.072
norm	Logit-sqrt	0.895	0.899	350	344.75	10.31	316	379	367.97	0.052
norm	Logit-sqrt	0.976	0.969	530	571.69	23.90	505	661	626.56	0.000
norm	Naïve	0.248	0.249	50	53.02	4.12	50	70	64.38	0.000
norm	Naïve	0.807	0.787	270	288.42	13.00	233	322	316.80	0.000
norm	Naïve	0.877	0.849	330	357.51	8.89	323	385	380.48	0.000
norm	Naïve	0.895	0.870	350	374.76	8.81	346	402	396.48	0.000
norm	Naïve	0.976	0.969	530	548.58	16.91	489	599	582.12	0.000
norm	Probit	0.248	0.343	50	5.83	7.24	1	42	24.25	0.988
norm	Probit	0.807	0.781	270	287.53	6.85	270	309	301.44	0.000
norm	Probit	0.877	0.863	330	342.61	8.39	318	372	359.72	0.000
norm	Probit	0.895	0.885	350	359.64	9.02	333	393	378.04	0.001
norm	Probit	0.976	0.985	530	496.17	15.17	452	554	526.98	0.560
norm	Probit-sqrt	0.248	0.240	50	52.74	6.49	32	75	64.65	0.007

Dist	Model	$\hat{\rho }_{\text {ref},N_\alpha }$	$\hat{\rho }_{N_\alpha }$	$N_\alpha$	$M(\hat{N}_{\alpha , \star })$	$SD(\hat{N}_{\alpha , \star })$	$min(\hat{N}_{\alpha , \star })$	$max(\hat{N}_{\alpha , \star })$	$M(\hat{N}_{\alpha , \text {lb}})$	Type I
norm	Probit-sqrt	0.807	0.808	270	269.72	6.84	251	292	284.00	0.021
norm	Probit-sqrt	0.877	0.877	330	330.37	8.79	306	360	349.33	0.019
norm	Probit-sqrt	0.895	0.895	350	350.39	9.63	323	384	371.31	0.019
norm	Probit-sqrt	0.976	0.976	530	532.08	19.43	477	604	574.91	0.020
norm	Wald	0.248	0.241	50	52.46	6.73	30	75	64.56	0.010
norm	Wald	0.807	0.808	270	269.75	6.84	251	292	284.03	0.021
norm	Wald	0.877	0.877	330	330.38	8.79	306	360	349.30	0.022
norm	Wald	0.895	0.895	350	350.37	9.62	323	384	371.22	0.021
norm	Wald	0.976	0.976	530	531.98	19.37	477	603	574.51	0.021
VM	Logit	0.214	0.284	50	9.14	9.44	1	48	30.92	0.939
VM	Logit	0.729	0.713	270	280.02	7.09	260	303	293.92	0.000
VM	Logit	0.811	0.804	330	336.25	8.35	314	364	353.18	0.001
VM	Logit	0.832	0.829	350	353.79	8.91	330	386	371.97	0.006
VM	Logit	0.949	0.956	530	511.61	15.63	465	577	544.58	0.200
VM	Logit	0.975	0.980	630	599.54	19.90	540	684	641.86	0.300
VM	Logit-sqrt	0.214	0.196	50	56.76	6.89	29	79	69.66	0.003
VM	Logit-sqrt	0.729	0.741	270	262.72	7.21	242	286	276.94	0.150
VM	Logit-sqrt	0.811	0.819	330	323.56	8.92	301	353	342.33	0.086
VM	Logit-sqrt	0.832	0.839	350	343.79	9.70	319	378	364.52	0.079
VM	Logit-sqrt	0.949	0.942	530	554.06	21.03	494	646	601.71	0.000
VM	Logit-sqrt	0.975	0.965	630	692.90	30.08	608	826	761.61	0.000
VM	Naïve	0.214	0.214	50	53.27	4.41	50	72	66.16	0.000
VM	Naïve	0.729	0.713	270	282.99	12.72	232	319	311.11	0.000
VM	Naïve	0.811	0.786	330	351.40	9.27	319	389	375.66	0.000
VM	Naïve	0.832	0.810	350	369.15	9.31	340	407	392.45	0.000
VM	Naïve	0.949	0.939	530	545.95	15.78	499	598	580.75	0.000
VM	Probit	0.214	0.303	50	3.64	5.66	1	38	20.24	0.993
VM	Probit	0.729	0.700	270	288.19	7.12	267	311	301.85	0.000
VM	Probit	0.811	0.791	330	345.76	8.08	323	372	361.74	0.000
VM	Probit	0.832	0.817	350	363.06	8.56	339	392	380.10	0.000
VM	Probit	0.949	0.960	530	504.32	14.02	462	561	533.03	0.420
VM	Probit	0.975	0.987	630	572.33	17.12	521	642	607.60	0.867
VM	Probit-sqrt	0.214	0.206	50	53.28	6.91	25	76	66.24	0.003
VM	Probit-sqrt	0.729	0.731	270	269.10	7.13	248	292	282.93	0.035
VM	Probit-sqrt	0.811	0.811	330	330.48	8.59	308	358	348.04	0.019
VM	Probit-sqrt	0.832	0.833	350	350.14	9.25	325	382	369.31	0.020
VM	Probit-sqrt	0.949	0.948	530	532.14	18.07	480	609	571.70	0.011
VM	Probit-sqrt	0.975	0.974	630	633.34	24.08	564	738	686.46	0.012
VM	Wald	0.214	0.207	50	52.86	7.24	21	76	66.12	0.003
VM	Wald	0.729	0.731	270	269.15	7.12	248	292	282.98	0.034
VM	Wald	0.811	0.811	330	330.50	8.59	308	358	348.04	0.020
VM	Wald	0.832	0.833	350	350.14	9.24	325	382	369.31	0.022
VM	Wald	0.949	0.948	530	532.00	17.98	480	608	571.32	0.011
VM	Wald	0.975	0.974	630	633.11	23.94	564	736	685.86	0.013

*Note.* Dist = distributional condition (norm = normal distribution, VM = Vale-Maurelli), Model = power model, $\hat{\rho }_{\text {ref}, N_\alpha }$ = computed reference power, $\hat{\rho }_{N_\alpha }$, $N_\alpha$ = reference required sample size, $M(\cdot )$ = mean, $SD(\cdot )$ = standard deviation, $min(\cdot )$ = minimum, $max(\cdot )$ = maximum, $\hat{N}_{\alpha , \star }$ = computed required sample size, $\hat{N}_{\alpha , \text {lb}}$ = computed required sample size using lower bound of power, Type I = Type I error rate of lower $\hat{N}_{\alpha , \text {lb}}$ being smaller than $N_\alpha$, $N_\alpha$ are selected for $\hat{\rho }_{\text {ref}, N_\alpha }$ to be close to .8 and .9 for the normal condition and to include the smallest and largest selected sample sizes

**Table 6:** Performance of the MSPE and alternative methods for linear SEM for $R=2000$ for LRT for selected reference sample sizes

Dist	Model	$\hat{\rho }_{\text {ref},N_\alpha }$	$\hat{\rho }_{N_\alpha }$	$N_\alpha$	$M(\hat{N}_{\alpha , \star })$	$SD(\hat{N}_{\alpha , \star })$	$min(\hat{N}_{\alpha , \star })$	$max(\hat{N}_{\alpha , \star })$	$M(\hat{N}_{\alpha , \text {lb}})$	Type I
norm	Logit	0.232	0.303	50	14.50	10.56	1	49	35.58	0.904
norm	Logit	0.802	0.791	270	277.29	6.83	259	298	291.39	0.000
norm	Logit	0.892	0.893	350	349.81	9.27	322	383	369.38	0.026
norm	Logit	0.976	0.980	530	510.37	16.88	456	575	546.10	0.196
norm	Logit-sqrt	0.232	0.211	50	56.48	6.25	33	76	68.00	0.001
norm	Logit-sqrt	0.802	0.814	270	261.80	6.90	243	282	276.46	0.170
norm	Logit-sqrt	0.892	0.897	350	344.71	10.14	315	379	367.48	0.056
norm	Logit-sqrt	0.976	0.969	530	568.83	23.36	495	657	622.32	0.000
norm	Naïve	0.232	0.232	50	53.00	4.06	50	71	63.93	0.000
norm	Naïve	0.802	0.782	270	288.60	12.81	236	323	316.57	0.000
norm	Naïve	0.892	0.866	350	374.86	8.63	343	403	396.29	0.000
norm	Naïve	0.976	0.968	530	548.05	16.49	483	597	580.77	0.000
norm	Probit	0.232	0.331	50	4.41	6.10	1	36	21.16	0.996
norm	Probit	0.802	0.775	270	287.98	6.78	270	308	301.68	0.000
norm	Probit	0.892	0.883	350	359.83	8.91	333	392	377.93	0.001
norm	Probit	0.976	0.984	530	495.42	14.90	448	552	525.65	0.589
norm	Probit-sqrt	0.232	0.229	50	51.47	6.35	28	71	63.23	0.013
norm	Probit-sqrt	0.802	0.802	270	270.23	6.76	252	290	284.29	0.016
norm	Probit-sqrt	0.892	0.892	350	350.52	9.50	323	384	371.05	0.016
norm	Probit-sqrt	0.976	0.976	530	530.54	19.04	470	603	572.39	0.023
norm	Wald	0.232	0.230	50	51.25	6.52	26	71	63.18	0.014
norm	Wald	0.802	0.802	270	270.26	6.76	252	290	284.31	0.016
norm	Wald	0.892	0.892	350	350.52	9.49	323	384	371.01	0.016
norm	Wald	0.976	0.976	530	530.44	19.00	470	602	572.09	0.021
VM	Logit	0.054	0.186	50	1.00	0.00	1	1	1.00	1.000
VM	Logit	0.671	0.634	270	287.99	6.09	267	307	300.07	0.000
VM	Logit	0.794	0.784	350	357.15	7.61	332	386	372.50	0.004
VM	Logit	0.934	0.950	530	498.85	13.00	458	547	525.63	0.632
VM	Logit	0.967	0.979	630	577.21	16.45	527	637	611.22	0.840
VM	Logit-sqrt	0.054	0.108	50	21.93	4.99	6	39	32.04	0.999
VM	Logit-sqrt	0.671	0.668	270	271.81	6.16	251	291	284.06	0.012
VM	Logit-sqrt	0.794	0.800	350	346.31	8.09	320	377	363.32	0.050
VM	Logit-sqrt	0.934	0.936	530	527.13	16.58	477	591	563.57	0.028
VM	Logit-sqrt	0.967	0.964	630	643.40	23.21	575	730	694.88	0.006
VM	Naïve	0.054	0.054	50	51.65	2.24	50	62	58.06	0.000
VM	Naïve	0.671	0.652	270	282.77	11.14	233	309	307.95	0.000
VM	Naïve	0.794	0.768	350	368.99	8.48	343	396	390.38	0.000
VM	Naïve	0.934	0.923	530	547.99	14.58	499	599	580.80	0.000
VM	Probit	0.054	0.195	50	1.00	0.00	1	1	1.00	1.000
VM	Probit	0.671	0.621	270	295.51	6.06	275	314	307.43	0.000
VM	Probit	0.794	0.769	350	366.89	7.31	342	393	381.45	0.000
VM	Probit	0.934	0.954	530	496.72	11.77	458	539	520.41	0.762
VM	Probit	0.967	0.987	630	558.87	14.27	514	608	587.70	0.992
VM	Probit-sqrt	0.054	0.107	50	26.09	5.17	9	43	36.34	0.992
VM	Probit-sqrt	0.671	0.657	270	277.37	6.06	257	296	289.36	0.001
VM	Probit-sqrt	0.794	0.790	350	353.47	7.77	328	382	369.53	0.006
VM	Probit-sqrt	0.934	0.941	530	515.68	14.58	470	571	546.84	0.136
VM	Probit-sqrt	0.967	0.973	630	604.10	19.14	547	674	645.26	0.245
VM	Wald	0.054	0.107	50	25.86	5.36	7	43	36.15	0.992
VM	Wald	0.671	0.657	270	277.37	6.06	257	296	289.42	0.001
VM	Wald	0.794	0.790	350	353.46	7.77	328	382	369.76	0.006
VM	Wald	0.934	0.941	530	515.65	14.57	470	571	547.25	0.132
VM	Wald	0.967	0.973	630	604.06	19.11	547	674	645.67	0.243

*Note.* Dist = distributional condition (norm = normal distribution, VM = Vale-Maurelli), Model = power model, $\hat{\rho }_{\text {ref}, N_\alpha }$ = computed reference power, $\hat{\rho }_{N_\alpha }$, $N_\alpha$ = reference required sample size, $M(\cdot )$ = mean, $SD(\cdot )$ = standard deviation, $min(\cdot )$ = minimum, $max(\cdot )$ = maximum, $\hat{N}_{\alpha , \star }$ = computed required sample size, $\hat{N}_{\alpha , \text {lb}}$ = computed required sample size using lower bound of power, type I = type I error rate of lower $\hat{N}_{\alpha , \text {lb}}$ being smaller than $N_\alpha$, $N_\alpha$ are selected for $\hat{\rho }_{\text {ref}, N_\alpha }$ to be close to .8 and .9 for the normal condition and to include the smallest and largest selected sample sizes

**Table 7:** Performance of the MSPE and alternative methods for QISEM for $R=2000$ for one-sided *z*-test for selected reference sample sizes

Dist	Model	$\hat{\rho }_{\text {ref},N_\alpha }$	$\hat{\rho }_{N_\alpha }$	$N_\alpha$	$M(\hat{N}_{\alpha , \star })$	$SD(\hat{N}_{\alpha , \star })$	$min(\hat{N}_{\alpha , \star })$	$max(\hat{N}_{\alpha , \star })$	$M(\hat{N}_{\alpha , \text {lb}})$	Type I
norm	Logit	0.360	0.408	150	102.53	25.28	1	178	148.95	0.490
norm	Logit	0.637	0.624	350	362.64	14.90	311	410	391.89	0.002
norm	Logit	0.809	0.800	550	564.13	15.08	515	607	595.02	0.002
norm	Logit	0.905	0.906	750	748.97	22.05	688	819	794.88	0.026
norm	Logit	0.934	0.937	850	838.75	26.43	762	926	894.13	0.059
norm	Logit	0.982	0.986	1200	1153.06	43.50	1015	1299	1245.56	0.176
norm	Logit-sqrt	0.360	0.335	150	164.20	14.09	109	210	191.53	0.003
norm	Logit-sqrt	0.637	0.643	350	345.56	12.72	302	387	370.54	0.040
norm	Logit-sqrt	0.809	0.821	550	532.26	15.02	484	574	563.70	0.185
norm	Logit-sqrt	0.905	0.909	750	739.00	24.26	672	816	792.50	0.058
norm	Logit-sqrt	0.934	0.934	850	851.64	30.92	766	954	920.75	0.022
norm	Logit-sqrt	0.982	0.976	1200	1309.19	62.72	1117	1522	1452.99	0.000
norm	Naïve	0.360	0.356	150	155.58	13.71	140	206	188.09	0.012
norm	Naïve	0.637	0.593	350	387.34	11.92	352	448	418.29	0.000
norm	Naïve	0.809	0.798	550	572.09	33.86	487	677	640.30	0.002
norm	Naïve	0.905	0.885	750	796.38	21.90	719	873	847.79	0.000
norm	Naïve	0.934	0.928	850	871.55	36.16	794	1014	964.60	0.004
norm	Naïve	0.982	0.980	1200	1172.60	23.41	1109	1236	1227.79	0.040
norm	Probit	0.360	0.437	150	65.76	28.56	1	150	118.88	0.901
norm	Probit	0.637	0.624	350	363.90	16.62	304	415	396.18	0.002
norm	Probit	0.809	0.786	550	584.39	15.10	534	630	614.68	0.000
norm	Probit	0.905	0.898	750	767.60	20.90	708	835	809.71	0.004
norm	Probit	0.934	0.934	850	849.33	24.55	776	931	899.03	0.036
norm	Probit	0.982	0.990	1200	1102.34	37.44	980	1229	1179.16	0.685
norm	Probit-sqrt	0.360	0.358	150	151.35	14.86	94	200	180.32	0.015
norm	Probit-sqrt	0.637	0.637	350	349.72	13.53	303	393	376.32	0.020
norm	Probit-sqrt	0.809	0.810	550	549.35	14.87	500	593	579.78	0.028
norm	Probit-sqrt	0.905	0.905	750	749.50	22.52	686	822	797.37	0.024
norm	Probit-sqrt	0.934	0.934	850	848.82	27.79	770	943	908.66	0.031
norm	Probit-sqrt	0.982	0.983	1200	1195.61	49.71	1039	1368	1305.25	0.031
norm	Wald	0.360	0.359	150	150.80	15.34	87	200	180.09	0.019

Dist	Model	$\hat{\rho }_{\text {ref},N_\alpha }$	$\hat{\rho }_{N_\alpha }$	$N_\alpha$	$M(\hat{N}_{\alpha , \star })$	$SD(\hat{N}_{\alpha , \star })$	$min(\hat{N}_{\alpha , \star })$	$max(\hat{N}_{\alpha , \star })$	$M(\hat{N}_{\alpha , \text {lb}})$	Type I
norm	Wald	0.637	0.637	350	349.88	13.49	303	393	376.35	0.020
norm	Wald	0.809	0.810	550	549.46	14.86	501	593	579.90	0.027
norm	Wald	0.905	0.905	750	749.48	22.51	686	822	797.24	0.024
norm	Wald	0.934	0.934	850	848.74	27.73	770	942	908.28	0.031
norm	Wald	0.982	0.983	1200	1195.17	49.42	1039	1367	1303.79	0.031
VM	Logit	0.527	0.559	150	128.50	21.75	59	188	166.75	0.169
VM	Logit	0.821	0.815	350	357.28	13.40	299	402	384.04	0.008
VM	Logit	0.938	0.938	550	550.08	20.57	469	628	593.85	0.026
VM	Logit	0.980	0.981	750	736.04	32.88	633	852	807.32	0.067
VM	Logit	0.989	0.990	850	831.59	39.78	715	967	918.20	0.069
VM	Logit-sqrt	0.527	0.495	150	162.82	12.78	126	199	186.94	0.000
VM	Logit-sqrt	0.821	0.835	350	336.13	12.18	284	376	360.54	0.174
VM	Logit-sqrt	0.938	0.944	550	530.88	20.39	449	612	577.20	0.109
VM	Logit-sqrt	0.980	0.979	750	760.88	37.23	643	903	849.79	0.011
VM	Logit-sqrt	0.989	0.986	850	895.16	48.51	756	1075	1012.33	0.000
VM	Naïve	0.527	0.516	150	161.11	15.91	140	209	194.94	0.006
VM	Naïve	0.821	0.743	350	419.20	9.44	390	455	442.30	0.000
VM	Naïve	0.938	0.930	550	589.74	47.35	492	717	675.78	0.003
VM	Naïve	0.980	0.967	750	817.89	23.86	749	934	880.84	0.000
VM	Naïve	0.989	0.986	850	888.25	56.60	812	1099	1014.20	0.007
VM	Probit	0.527	0.598	150	89.96	28.85	1	169	138.38	0.673
VM	Probit	0.821	0.805	350	369.69	14.88	305	416	398.74	0.001
VM	Probit	0.938	0.929	550	572.85	21.18	490	645	614.49	0.002
VM	Probit	0.980	0.981	750	738.84	32.47	642	838	802.85	0.075
VM	Probit	0.989	0.991	850	814.80	38.18	702	926	890.31	0.166
VM	Probit-sqrt	0.527	0.532	150	147.98	14.79	105	189	175.62	0.026
VM	Probit-sqrt	0.821	0.822	350	349.10	13.16	293	391	375.00	0.029
VM	Probit-sqrt	0.938	0.939	550	549.03	20.10	467	624	591.85	0.030
VM	Probit-sqrt	0.980	0.980	750	745.98	33.81	639	864	820.80	0.036
VM	Probit-sqrt	0.989	0.989	850	846.22	41.94	726	987	939.94	0.035
VM	Wald	0.527	0.532	150	147.57	15.22	102	189	175.32	0.031
VM	Wald	0.821	0.822	350	349.26	13.10	293	391	375.10	0.028
VM	Wald	0.938	0.939	550	549.09	20.12	467	624	591.97	0.029
VM	Wald	0.980	0.980	750	745.84	33.71	639	864	820.21	0.035
VM	Wald	0.989	0.989	850	845.95	41.75	726	987	938.89	0.034

*Note.* Dist = distributional condition (norm = normal distribution, VM = Vale-Maurelli), Model = power model, $\hat{\rho }_{\text {ref}, N_\alpha }$ = computed reference power, $\hat{\rho }_{N_\alpha }$, $N_\alpha$ = reference required sample size, $M(\cdot )$ = mean, $SD(\cdot )$ = standard deviation, $min(\cdot )$ = minimum, $max(\cdot )$ = maximum, $\hat{N}_{\alpha , \star }$ = computed required sample size, $\hat{N}_{\alpha , \text {lb}}$ = computed required sample size using lower bound of power, type I = type I error rate of lower $\hat{N}_{\alpha , \text {lb}}$ being smaller than $N_\alpha$, $N_\alpha$ are selected for $\hat{\rho }_{\text {ref}, N_\alpha }$ to be close to .8 and .9 for the normal condition and to include the smallest and largest selected sample sizes

**Table 8:** Performance of the MSPE and alternative methods for QISEM for $R=2000$ for two-sided *z*-test for selected reference sample sizes

Dist	Model	$\hat{\rho }_{\text {ref},N_\alpha }$	$\hat{\rho }_{N_\alpha }$	$N_\alpha$	$M(\hat{N}_{\alpha , \star })$	$SD(\hat{N}_{\alpha , \star })$	$min(\hat{N}_{\alpha , \star })$	$max(\hat{N}_{\alpha , \star })$	$M(\hat{N}_{\alpha , \text {lb}})$	Type I
norm	Logit	0.254	0.309	150	82.45	26.83	2	175	131.96	0.761
norm	Logit	0.570	0.552	400	417.96	14.41	375	462	446.27	0.000
norm	Logit	0.812	0.807	700	709.99	17.18	649	756	744.63	0.007
norm	Logit	0.882	0.885	850	844.96	22.42	772	912	890.73	0.047
norm	Logit	0.900	0.904	900	889.35	24.39	813	964	939.30	0.068
norm	Logit	0.963	0.969	1200	1155.52	37.19	1050	1276	1232.90	0.223
norm	Logit-sqrt	0.254	0.239	150	161.42	14.68	119	217	189.60	0.000
norm	Logit-sqrt	0.570	0.575	400	396.27	12.88	360	437	421.28	0.045
norm	Logit-sqrt	0.812	0.821	700	685.75	18.23	620	736	723.94	0.117
norm	Logit-sqrt	0.882	0.884	850	846.31	25.86	762	921	901.90	0.032
norm	Logit-sqrt	0.900	0.899	900	902.82	28.95	811	988	965.46	0.019
norm	Logit-sqrt	0.963	0.955	1200	1279.96	52.51	1131	1446	1396.21	0.000
norm	Naïve	0.254	0.253	150	153.50	12.24	140	192	183.53	0.016
norm	Naïve	0.570	0.548	400	419.46	13.84	378	490	452.70	0.000
norm	Naïve	0.812	0.788	700	741.82	19.70	680	820	792.12	0.000
norm	Naïve	0.882	0.876	850	866.38	30.14	801	1002	944.16	0.006
norm	Naïve	0.900	0.896	900	918.59	44.79	826	1054	1015.16	0.013
norm	Naïve	0.963	0.960	1200	1175.38	22.94	1131	1241	1228.07	0.024
norm	Probit	0.254	0.327	150	57.19	28.25	1	156	110.32	0.954
norm	Probit	0.570	0.552	400	420.54	15.41	373	467	450.58	0.000
norm	Probit	0.812	0.795	700	728.51	16.53	671	774	761.23	0.000
norm	Probit	0.882	0.879	850	858.10	20.91	793	925	899.92	0.010
norm	Probit	0.900	0.900	900	898.64	22.53	831	973	943.85	0.035
norm	Probit	0.963	0.976	1200	1121.03	32.32	1028	1234	1186.85	0.640
norm	Probit-sqrt	0.254	0.251	150	152.99	14.83	110	209	181.70	0.011
norm	Probit-sqrt	0.570	0.571	400	399.46	13.23	361	441	425.28	0.027
norm	Probit-sqrt	0.812	0.813	700	699.55	17.46	638	747	735.28	0.022
norm	Probit-sqrt	0.882	0.882	850	850.91	23.81	774	924	900.78	0.024
norm	Probit-sqrt	0.900	0.899	900	901.33	26.30	820	985	956.71	0.024
norm	Probit-sqrt	0.963	0.963	1200	1203.58	43.31	1080	1351	1296.91	0.027
norm	Wald	0.254	0.251	150	152.75	15.05	108	209	181.63	0.011
norm	Wald	0.570	0.571	400	399.52	13.20	361	441	425.31	0.028
norm	Wald	0.812	0.813	700	699.56	17.47	638	747	735.29	0.023
norm	Wald	0.882	0.882	850	850.90	23.82	774	924	900.64	0.025
norm	Wald	0.900	0.899	900	901.30	26.26	820	984	956.52	0.023
norm	Wald	0.963	0.963	1200	1203.44	43.22	1080	1350	1296.43	0.026
VM	Logit	0.434	0.475	150	120.28	21.91	44	179	159.23	0.277
VM	Logit	0.799	0.789	400	411.18	13.08	364	447	437.20	0.005
VM	Logit	0.952	0.953	700	693.18	23.94	619	763	744.77	0.046
VM	Logit	0.977	0.979	850	832.60	32.02	740	935	902.38	0.071
VM	Logit-sqrt	0.434	0.404	150	163.07	12.75	122	200	187.02	0.000
VM	Logit-sqrt	0.799	0.813	400	385.72	12.27	342	421	410.17	0.194
VM	Logit-sqrt	0.952	0.953	700	692.32	25.93	614	768	752.60	0.035
VM	Logit-sqrt	0.977	0.975	850	876.92	38.44	769	1004	968.41	0.003
VM	Naïve	0.434	0.424	150	158.57	14.42	140	212	189.24	0.004
VM	Naïve	0.799	0.740	400	447.04	10.11	417	481	470.16	0.000
VM	Naïve	0.952	0.931	700	772.41	21.52	699	843	822.28	0.000
VM	Naïve	0.977	0.973	850	877.66	42.13	813	1051	988.60	0.007
VM	Probit	0.434	0.513	150	80.56	27.56	1	160	128.22	0.832
VM	Probit	0.799	0.777	400	426.81	14.10	376	465	454.19	0.000
VM	Probit	0.952	0.949	700	709.84	23.59	638	776	757.11	0.014
VM	Probit	0.977	0.981	850	827.88	30.37	737	920	889.24	0.113
VM	Probit-sqrt	0.434	0.438	150	148.25	14.17	103	189	174.85	0.026
VM	Probit-sqrt	0.799	0.799	400	400.54	12.89	355	437	425.76	0.028
VM	Probit-sqrt	0.952	0.952	700	698.71	24.13	624	768	751.55	0.027
VM	Probit-sqrt	0.977	0.978	850	847.55	33.52	750	956	922.50	0.026
VM	Wald	0.434	0.438	150	148.00	14.43	100	189	174.71	0.026
VM	Wald	0.799	0.799	400	400.62	12.86	355	437	425.83	0.028
VM	Wald	0.952	0.952	700	698.66	24.12	624	768	751.38	0.027
VM	Wald	0.977	0.978	850	847.43	33.49	750	956	922.02	0.029

*Note.* Dist = distributional condition (norm = normal distribution, VM = Vale-Maurelli), Model = power model, $\hat{\rho }_{\text {ref}, N_\alpha }$ = computed reference power, $\hat{\rho }_{N_\alpha }$, $N_\alpha$ = reference required sample size, $M(\cdot )$ = mean, $SD(\cdot )$ = standard deviation, $min(\cdot )$ = minimum, $max(\cdot )$ = maximum, $\hat{N}_{\alpha , \star }$ = computed required sample size, $\hat{N}_{\alpha , \text {lb}}$ = computed required sample size using lower bound of power, type I = type I error rate of lower $\hat{N}_{\alpha , \text {lb}}$ being smaller than $N_\alpha$, $N_\alpha$ are selected for $\hat{\rho }_{\text {ref}, N_\alpha }$ to be close to .8 and .9 for the normal condition and to include the smallest and largest selected sample sizes

**Table 9:** Performance of the MSPE and alternative methods for QISEM for $R=2000$ for LRT for selected reference sample sizes

Dist	Model	$\hat{\rho }_{\text {ref},N_\alpha }$	$\hat{\rho }_{N_\alpha }$	$N_\alpha$	$M(\hat{N}_{\alpha , \star })$	$SD(\hat{N}_{\alpha , \star })$	$min(\hat{N}_{\alpha , \star })$	$max(\hat{N}_{\alpha , \star })$	$M(\hat{N}_{\alpha , \text {lb}})$	Type I
norm	Logit	0.104	0.198	150	7.49	11.90	1	80	41.67	1.000
norm	Logit	0.776	0.761	700	718.54	14.92	663	764	747.58	0.001
norm	Logit	0.809	0.801	750	761.45	16.21	703	812	793.17	0.005
norm	Logit	0.861	0.865	850	843.09	19.06	780	903	880.76	0.062
norm	Logit	0.899	0.911	950	922.38	22.20	854	992	966.55	0.252
norm	Logit	0.957	0.970	1200	1119.73	30.73	1027	1212	1181.55	0.692
norm	Logit-sqrt	0.104	0.133	150	121.80	13.68	70	170	147.55	0.555
norm	Logit-sqrt	0.776	0.780	700	695.09	15.55	636	743	726.45	0.054
norm	Logit-sqrt	0.809	0.813	750	743.46	17.26	681	798	778.71	0.057
norm	Logit-sqrt	0.861	0.866	850	840.02	21.35	769	909	884.34	0.070
norm	Logit-sqrt	0.899	0.903	950	939.49	26.13	858	1023	994.41	0.059
norm	Logit-sqrt	0.957	0.956	1200	1211.13	41.10	1086	1337	1298.93	0.015
norm	Naïve	0.104	0.107	150	149.60	7.54	140	173	167.93	0.017
norm	Naïve	0.776	0.745	700	743.64	16.65	695	801	787.44	0.000
norm	Naïve	0.809	0.781	750	789.59	17.08	744	853	831.34	0.000
norm	Naïve	0.861	0.853	850	866.38	26.68	813	973	936.27	0.003
norm	Naïve	0.899	0.889	950	984.80	42.83	862	1098	1071.84	0.002
norm	Naïve	0.957	0.953	1200	1176.98	20.14	1133	1238	1225.11	0.015
norm	Probit	0.104	0.209	150	5.70	10.00	1	77	36.44	1.000
norm	Probit	0.776	0.745	700	737.87	14.31	684	780	765.50	0.000
norm	Probit	0.809	0.786	750	780.74	15.44	724	827	810.67	0.000
norm	Probit	0.861	0.855	850	859.52	17.89	800	913	894.41	0.006
norm	Probit	0.899	0.908	950	932.30	20.45	869	993	972.37	0.154
norm	Probit	0.957	0.977	1200	1097.55	26.92	1019	1174	1150.79	0.943
norm	Probit-sqrt	0.104	0.135	150	122.74	13.38	73	171	147.96	0.546
norm	Probit-sqrt	0.776	0.769	700	709.71	14.94	653	755	739.50	0.007
norm	Probit-sqrt	0.809	0.804	750	757.63	16.46	697	809	790.73	0.010
norm	Probit-sqrt	0.861	0.861	850	849.75	19.90	783	913	890.31	0.027
norm	Probit-sqrt	0.899	0.903	950	939.50	23.74	866	1014	988.44	0.068
norm	Probit-sqrt	0.957	0.964	1200	1160.14	34.59	1057	1264	1232.43	0.211
norm	Wald	0.104	0.135	150	122.71	13.42	72	171	147.74	0.551
norm	Wald	0.776	0.769	700	709.71	14.94	653	755	739.88	0.006
norm	Wald	0.809	0.804	750	757.63	16.46	697	809	791.20	0.009
norm	Wald	0.861	0.861	850	849.75	19.90	783	913	890.94	0.025
norm	Wald	0.899	0.903	950	939.50	23.73	866	1014	989.21	0.062
norm	Wald	0.957	0.964	1200	1160.13	34.58	1057	1264	1233.44	0.202
VM	Logit	0.166	0.281	150	6.18	11.54	1	69	39.56	1.000
VM	Logit	0.805	0.779	700	741.28	17.20	682	796	776.28	0.000
VM	Logit	0.829	0.811	750	781.28	18.69	719	843	819.26	0.000
VM	Logit	0.868	0.865	850	858.17	21.84	791	934	902.54	0.016
VM	Logit-sqrt	0.166	0.208	150	116.99	14.37	62	160	144.33	0.649
VM	Logit-sqrt	0.805	0.796	700	716.50	18.21	651	774	755.02	0.004
VM	Logit-sqrt	0.829	0.824	750	762.21	20.24	692	827	805.18	0.009
VM	Logit-sqrt	0.868	0.867	850	854.07	24.76	774	939	906.98	0.018
VM	Naïve	0.166	0.164	150	152.38	9.35	140	179	174.10	0.009
VM	Naïve	0.805	0.775	700	753.41	21.18	693	821	805.46	0.000
VM	Naïve	0.829	0.804	750	794.89	21.69	733	869	846.10	0.000
VM	Naïve	0.868	0.862	850	870.04	35.79	798	1004	968.70	0.007
VM	Probit	0.166	0.298	150	2.35	5.51	1	49	22.46	1.000
VM	Probit	0.805	0.765	700	762.18	16.47	708	815	795.54	0.000
VM	Probit	0.829	0.798	750	801.79	17.74	746	861	837.68	0.000
VM	Probit	0.868	0.856	850	875.67	20.44	812	948	916.97	0.001
VM	Probit-sqrt	0.166	0.218	150	111.29	14.34	58	153	138.65	0.781
VM	Probit-sqrt	0.805	0.787	700	732.59	17.43	673	787	769.05	0.000
VM	Probit-sqrt	0.829	0.815	750	777.28	19.16	714	840	817.47	0.001
VM	Probit-sqrt	0.868	0.863	850	864.14	23.02	793	945	912.65	0.008
VM	Wald	0.166	0.218	150	110.94	14.64	53	153	138.46	0.786
VM	Wald	0.805	0.787	700	732.56	17.42	672	787	769.87	0.000
VM	Wald	0.829	0.815	750	777.22	19.14	714	840	818.46	0.000
VM	Wald	0.868	0.863	850	864.05	22.97	793	944	913.90	0.007

*Note.* Dist = distributional condition (norm = normal distribution, VM = Vale-Maurelli), Model = power model, $\hat{\rho }_{\text {ref}, N_\alpha }$ = computed reference power, $\hat{\rho }_{N_\alpha }$, $N_\alpha$ = reference required sample size, $M(\cdot )$ = mean, $SD(\cdot )$ = standard deviation, $min(\cdot )$ = minimum, $max(\cdot )$ = maximum, $\hat{N}_{\alpha , \star }$ = computed required sample size, $\hat{N}_{\alpha , \text {lb}}$ = computed required sample size using lower bound of power, type I = type I error rate of lower $\hat{N}_{\alpha , \text {lb}}$ being smaller than $N_\alpha$, $N_\alpha$ are selected for $\hat{\rho }_{\text {ref}, N_\alpha }$ to be close to .8 and .9 for the normal condition and to include the smallest and largest selected sample sizes

## Appendix F: Information on R-packages used in the simulation

All empirical analyses were done in R (R Core Team, 2023). We used R-base and stats functions and mvtnorm (Genz & Bretz, 2009) and semTools (Jorgensen et al., 2020) for model simulation, and lavaan (Rosseel, 2012) for data handling and the fitting of SEM. For the two-sided version of the probit regression we used numerical derivation of the numDeriv package (Gilbert & Varadhan, 2019) to model uncertainty in power estimation. Parallelization is done via the pbapply package (Solymos & Zawadzki, 2023), and ggplot (Wickham, 2016) and ggpubr (Kassambara, 2023) is called to plot results.

We further utilized the papaja package for easy table handling (Aust & Barth, 2022).

Power for linear models was derived using semPower (Moshagen & Bader, 2024).

Table 10 shows the corresponding package versions as well as the package versions of implicitly loaded packages. The version number corresponding to the base package corresponds to the R version used.

**Table 10:** R Packages

Package	Version
base	4.4.0
datasets	4.4.0
ggplot2	3.5.1
ggpubr	0.6.0
graphics	4.4.0
grDevices	4.4.0
lavaan	0.6.18
methods	4.4.0
mvtnorm	1.2.5
numDeriv	2016.8.1.1
papaja	0.1.2
parallel	4.4.0
pbapply	1.7.2
psych	2.4.3
semPower	2.1.0
semTools	0.5.6
stats	4.4.0
stringr	1.5.1
tinylabels	0.2.4
utils	4.4.0

*Note.* Implicitly loaded packages are also displayed

## Code Availability

All code is available at https://osf.io/6qh4u/.
